# Restorative streetscapes in high-density urban tourism: environmental archetypes, visual attention, and psychophysiological responses

**DOI:** 10.3389/fpubh.2026.1981997

**Published:** 2026-10-22

**Authors:** Xinliang Wang, Yafei Zhao, Xiang Xu, Xiaoju Li, Jingyuan Cheng

**Affiliations:** 1 School of Economics and Management, Huainan Vocational and Technical College, Huainan, Anhui, China; 2 School of Architecture, Tianjin University, Tianjin, China; 3 School of Design and Architecture, Zhejiang University of Technology, Hangzhou, China; 4 School of Architecture, Harbin Institute of Technology (Shenzhen), Shenzhen, China

**Keywords:** environmental archetype, high-density tourist spaces, multimodal psychophysiological response, perceived restorativeness, streetscape visual environment

## Abstract

In high-density tourist districts, visitors are continuously exposed to the visual environments of surrounding streets, forecourts, and small public spaces before reaching an attraction, and these transitional spaces may influence short-term restorative states. Existing research has typically examined urban-scale streetscape characteristics and human responses under experimental conditions separately, with limited provision of a reproducible procedure for selecting experimental stimuli from large environmental samples. Taking the high-density urban core around Hong Kong’s Victoria Harbour as a case study, this study identified 66 tourism-related sites and acquired four fixed-direction streetscape images for each site, yielding 264 images in total. A 66 × 11 objective environmental matrix was constructed from five GIS-based environmental indicators and six streetscape-image proxy indicators. Principal component analysis (PCA) summarized the dominant environmental gradients, and k = 6 K-means was retained as a pragmatic six-group sampling solution aligned with the pre-specified six-scene experimental capacity rather than as a uniquely optimal or stable urban typology. Forty-six of 48 registered adults contributed 108 focal-scene exposures. After BH-FDR correction, significant differences among the six scenes were observed in perceived restorativeness, the proportion of fixations on natural areas of interest (AOIs), gaze entropy, changes in EEG alpha-minus-theta, mean EDA skin conductance level (SCL), and HRV RMSSD, whereas sustained-attention accuracy showed no significant scene effect. Oil Street Art Space exhibited the highest perceived restorativeness (5.69/7), the highest proportion of fixations on natural AOIs (51.4%), and the largest increases in EEG (+2.68) and HRV RMSSD (+11.37 ms), while Western Market showed the lowest restorativeness (4.17/7), the lowest natural-AOI fixation share (31.2%), and the smallest EEG increase (+1.93). Sensitivity analyses showed no evidence of scene-by-order interaction for perceived restorativeness, natural-AOI fixation, gaze entropy, or SART accuracy, but EEG, EDA, and HRV were more order-sensitive; the principal subjective, attentional, EEG, and HRV contrasts remained evident when analysis was restricted to each participant’s earliest focal-scene exposure. These findings therefore support scene-configuration contrasts under short-term static visual exposure. Building on the environmental structure of the 66 sites and the multimodal responses to the six experimental scenes, the study further proposes an archetype-calibrated framework for mapping restorative potential in high-density tourist streetscapes, providing a spatial basis for identifying restorative opportunity nodes and priority intervention areas.

## 1 Introduction

In high-density tourist destinations, visitors’ environmental experience does not begin at the entrance to an attraction. Whether walking from a transport node to a destination, pausing briefly in a forecourt, or moving through commercial and heritage streets, visitors are continuously exposed to vegetation, water, sky, building façades, traffic, signage, crowds, and landmarks. These approach spaces differ from general urban streetscapes because exposure is purposeful and sequential: visitors are orienting toward a destination, interpreting place identity, making route decisions, and often moving through unfamiliar environments under time constraints. Consequently, restorative appraisal in tourist approach spaces may interact with landmark recognition, visual order, destination image, and the cumulative sensory experience of walking, rather than reflecting environmental naturalness alone. Large visitor-generated datasets from Hong Kong and Victoria Harbour likewise show that tourists’ sensory experience and destination image vary with waterfront context, length of stay, and the content of online reviews and social-media posts (1–3). Streets and public spaces surrounding attractions should therefore be examined as components of the tourism experience as well as urban environmental exposures.

Existing research provides evidence for this issue from two main directions. First, environmental psychology has shown that natural environments and urban spaces with restorative qualities can influence mood, attention, and stress recovery (4–18). Second, streetscape and urban-visual research has developed urban-scale quantitative approaches for measuring greenery, blue space, sky openness, urban form, and image structure from a pedestrian perspective (19–29). Yet these two research streams remain only weakly integrated: the former typically relies on a small number of experimental scenes, whereas the latter focuses on large-scale environmental measurement. Recent reviews have likewise identified eye-level natural exposure, data quality, geographic diversity, and causal identification as continuing limitations in the field (30–32). A research design capable of linking urban-scale environmental measurement with controlled human-subject experiments is therefore needed.

The central methodological challenge is how to select a limited number of experimental scenes from a large urban streetscape sample while still capturing the major dimensions of environmental variation. If images are selected merely for convenience or according to researchers’ subjective judgment, the representativeness and position of the chosen stimuli within the broader urban environment become difficult to establish. Conversely, selecting scenes retrospectively because they produce better participant responses introduces outcome-driven selection bias. In this study, scene selection was therefore completed before analysis of human responses and was based on pre-specified sites, consistently derived environmental indicators, and unsupervised grouping, thereby reducing urban-scale environmental variation to an experimentally feasible set of scenes.

Within this framework, PCA and K-means serve upstream functions in environmental organization and scene selection. PCA reduces the 11 correlated indicators to a small number of dominant environmental gradients, while K-means identifies environmental archetypes characterized by distinct multivariate combinations. This procedure increases the transparency of scene selection and positions the six experimental scenes explicitly within the broader urban environmental sample.

The tourism context further illustrates why interpretation should be conducted at the level of complete scenes. Experiences of streets and public spaces may be influenced not only by visible natural content but also by landmark visibility, historic architecture, local distinctiveness, route legibility, and visual order. Recent city-walk research identifies visual order as an important trigger within dynamic cognitive-affective appraisal during walking tourism (33), while studies based on visitor-generated data in Hong Kong show that destination image and sensory experience vary across visitor groups and spatial contexts (1–3). Museum and heritage environments may affect restorativeness and place experience through pathways other than simply increasing greenery (34–37). At the same time, non-visual factors such as sound, thermal conditions, humidity, and crowding influence urban well-being (38–44). Static photographs therefore enable controlled comparisons of visual scene configurations, and the experiment might explore future scenarios of multisensory, sequential tourist experience.

The two sides of Victoria Harbour provide a suitable setting in which to test this research pathway. Within a relatively compact, high-density urban area, historic buildings, museums, commercial streets, transport facilities, landscaped green spaces, and waterfront public spaces are highly concentrated while exhibiting marked variation in built intensity and visual environment. Using official geospatial sources, this study identified 66 tourism-related analytical sites and constructed an 11-indicator environmental matrix from 264 streetscape images and GIS context. PCA and K-means were then used to identify six environmental archetypes and, on that basis, six field-photographed experimental scenes. Finally, subjective evaluation, eye tracking, EEG, EDA, and HRV were used to compare short-term restoration-related responses across the six scenes.

Four research questions were formulated around this analytical chain. RQ1: What forms of environmental heterogeneity are present across the 66 tourism-related sites in terms of GIS context and streetscape visual proxy indicators? RQ2: How can PCA and a pragmatic six-group K-means sampling solution organize the 66 sites for transparent six-scene selection, and where do the six field-photographed scenes lie within this environmental space? RQ3: After accounting for repeated observations and presentation order, do the six complete scenes differ in perceived restorativeness and short-term cognitive performance? RQ4: Do the six complete scenes elicit different patterns of visual attention, EEG, EDA, and HRV, and how robust are these contrasts to trial order, modality-specific missingness, and first-focal-exposure sensitivity analyses? Table 1 summarizes the evidence sources and analytical purpose associated with each research question.

**Table 1:** Research questions, primary evidence and analytical purpose.

Question	Primary evidence	Analytical purpose
RQ1	66 official sites; 264 validated street views; eleven objective indicators	Quantify site-level environmental heterogeneity and indicator distributions
RQ2	66 × 11 standardized matrix; PCA; k = 6 K-means; centroid matching	Define environmental archetypes and document how the six photographed candidates relate to them
RQ3	Six-scene perceived-restoration ratings and sustained-attention task	Estimate fixed-scene differences in short-term restorative appraisal and cognitive performance
RQ4	Eye tracking, EEG, EDA, HRV; standardized scene profiles; complete-case sensitivity	Estimate construct-specific multimodal scene differences and partial cross-modal convergence

This study makes four main contributions. First, it establishes a traceable selection chain from 66 urban sites and 264 streetscape images to six experimental scenes, thereby reducing the risks associated with convenience-based or outcome-driven stimulus selection. Second, it integrates objective spatial context, subjective restorativeness, visual attention, EEG, and autonomic responses within a single framework while explicitly defining the interpretive scope of each evidence layer. Third, given the incomplete repeated-measures structure, it applies participant-level repeated-measures analyses, modality-specific valid sample sizes, order-by-scene and first-focal-exposure sensitivity checks, missingness reporting, and multiple-comparison control. Fourth, it shifts the focus from identifying a single “best” feature to comparing complete tourist streetscape configurations under controlled short-term visual exposure, and links the environmental archetypes with the six-scene multimodal response profiles to support an archetype-calibrated framework for mapping restorative potential (Figure 1).

**Figure 1:**
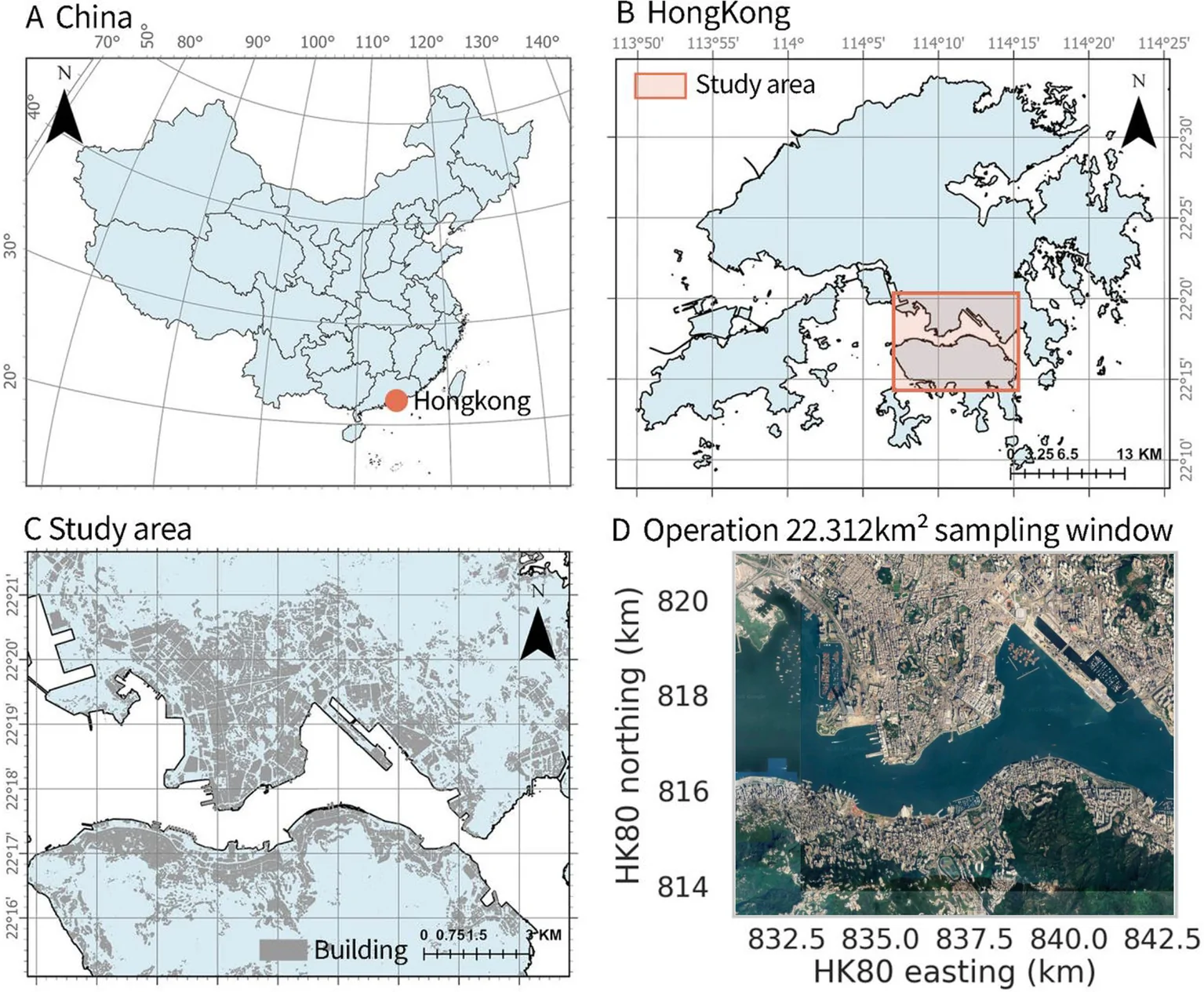
Victoria Harbour high-density urban core analytical context.

## 2 Literature review and theoretical framework

### 2.1 Restorative exposure, temporal responses, and contextualized evaluation

Attention Restoration Theory (ART) and Stress Recovery Theory (SRT) explain environmental restoration from the perspectives of cognitive-resource replenishment and stress response, respectively. ART proposes that experiences such as being away, fascination, extent/coherence, and compatibility can help alleviate directed-attention fatigue (4, 8, 9), whereas SRT emphasizes that environmental cues can alter affect and physiological arousal over relatively short time scales (5, 6). Field and experimental studies have shown that natural environments and urban spaces with restorative characteristics are associated with affective, attentional, and stress-related outcomes, although effect magnitude and onset depend on exposure duration, task type, population, and context (7, 10–18). Accordingly, this study expects different scenes to elicit differences across multiple response channels without requiring all measures to change synchronously on the same time scale.

For 60-s visual exposures, distinguishing the temporal sensitivity of different response measures is particularly important. Subjective states and autonomic responses may change within a short period, whereas sustained-attention accuracy may not show detectable differences over the same interval and may also be influenced by task difficulty and speed–accuracy trade-offs. A null effect in cognitive accuracy therefore cannot, by itself, be taken as evidence that restoration did not occur; it is more appropriately interpreted alongside subjective ratings, eye-tracking measures, and physiological responses. On this basis, perceived restorativeness, visual attention, neuro-autonomic responses, and cognitive performance are treated here as related but distinct outcome domains.

In tourist environments, restorative experience is also shaped by place meaning and route function. Natural content may contribute to comfort and restoration, while landmark visibility and route legibility may improve orientation, and heritage features and local distinctiveness may increase scene meaning and attractiveness (34–37). Visitor-generated evidence from Hong Kong further indicates that destination image varies with travel context and length of stay, and that waterfront sensory experience differs spatially and between tourists and residents (1–3). City-walk research similarly emphasizes a dynamic appraisal process in which visual order interacts with other environmental cues rather than acting as an isolated determinant (33). These factors often co-occur in high-density tourist districts. ART and SRT are therefore used here to interpret short-term restoration-related responses, whereas heritage, landmarks, visual order, and tourism functions are treated as contextual properties of complete scenes rather than as independently identified causal factors.

The experiment focuses on visual exposure. Standardized static photographs facilitate control of visual input but exclude walking, sound, thermal and humidity conditions, odor, route continuity, and dynamic crowding that characterize real tourist experiences (Figure 2). The project stimulus protocol also specifies that the experimental materials are static two-dimensional images; even when presented through VR/eye-tracking equipment, they should not be described as immersive virtual environments with panoramic geometry or three-dimensional interaction. Evidence relating to soundscape, noise, thermal comfort, and multisensory urban experience (38–44) therefore defines an important boundary on the external validity of the present study.

**Figure 2:**
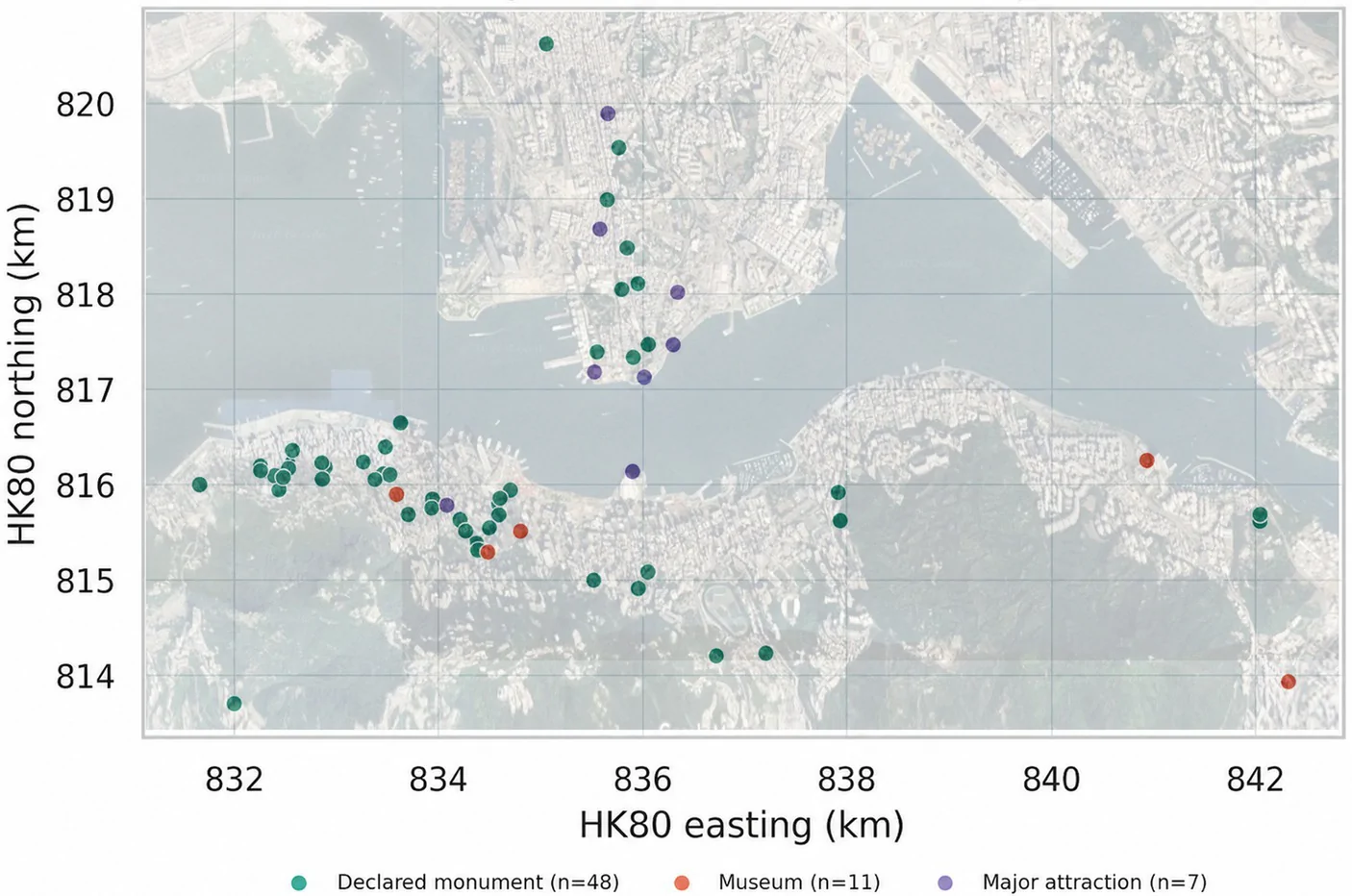
Distribution of analytical sites and focal experimental locations.

### 2.2 Objective streetscape context and eye-level visual measurement

Street-level imagery complements overhead-scale urban indicators because the greenery, blue space, and sky actually visible to pedestrians at eye level are not equivalent to remotely sensed land cover or administratively defined green-space provision (19–23, 30, 31, 45–47). It is also necessary to distinguish the meanings of different image-based measurements: color proxies quantify the proportion of pixels meeting pre-defined color rules, semantic segmentation estimates object classes, and eye-tracking data record where participants actually look. These approaches measure different constructs and should not be treated as interchangeable in the absence of validation.

GIS morphological indicators describe the spatial context within which streetscapes are formed. Building coverage, building height, and road-network intensity influence enclosure, sight distance, and openness, but they cannot directly determine which visual elements appear in a photograph. Accordingly, this study combines GIS morphological indicators with street-level visual indicators: the former characterize the built-environment context surrounding each attraction, whereas the latter characterize visible exposure from a pedestrian perspective. In a high-density subtropical city, this distinction also helps prevent morphological effects related to thermal conditions, air quality, or acoustic enclosure from being misinterpreted as purely visual effects (48–51).

Image-structure indicators likewise require clear construct boundaries. Luminance entropy describes the distribution of image information, while saliency measures indicate the potential of low-level visual features to attract attention (27–29); neither constitutes an esthetic or restorative rating. A historic façade and a commercial street may display similar statistical complexity yet evoke substantially different scene experiences. Recent reviews of urban visual perception similarly note the rapid development of computational-vision methods while emphasizing the continued need for construct validation and stronger causal interpretation (32).

Based on these distinctions, this study adopts a hierarchical measurement strategy. GIS indicators within 250 m characterize urban morphology and harbor proximity around each site; four quality-checked streetscape views per site represent visible visual exposure; standardized color and texture rules generate reproducible image proxies; PCA summarizes the dominant environmental gradients; and K-means provides a pragmatic grouping used to structure six-scene sampling. Each step is assigned only the role appropriate to its data level—for example, the proportion of blue pixels is not equated directly with visible water, green-color share is not equated with validated vegetation cover, and image saliency is not equated with participants’ actual gaze.

These image proxies remain susceptible to systematic error. Color rules may classify painted surfaces, signs, or other objects as green or blue and may confound blue sky with water; the strong blue–sky proxy correlation observed in the present data is consistent with such construct overlap. Vehicles, pedestrians, and temporary objects may also alter local visibility, and averaging across four directions may attenuate directional extremes. Accordingly, the original views in all four directions are retained, and color-derived measures are consistently described as proxy indicators throughout the manuscript. The SAM outputs shown for the experimental stimulus set in Figure 3 were used to support scene-level semantic annotation/AOI definition.

**Figure 3:**
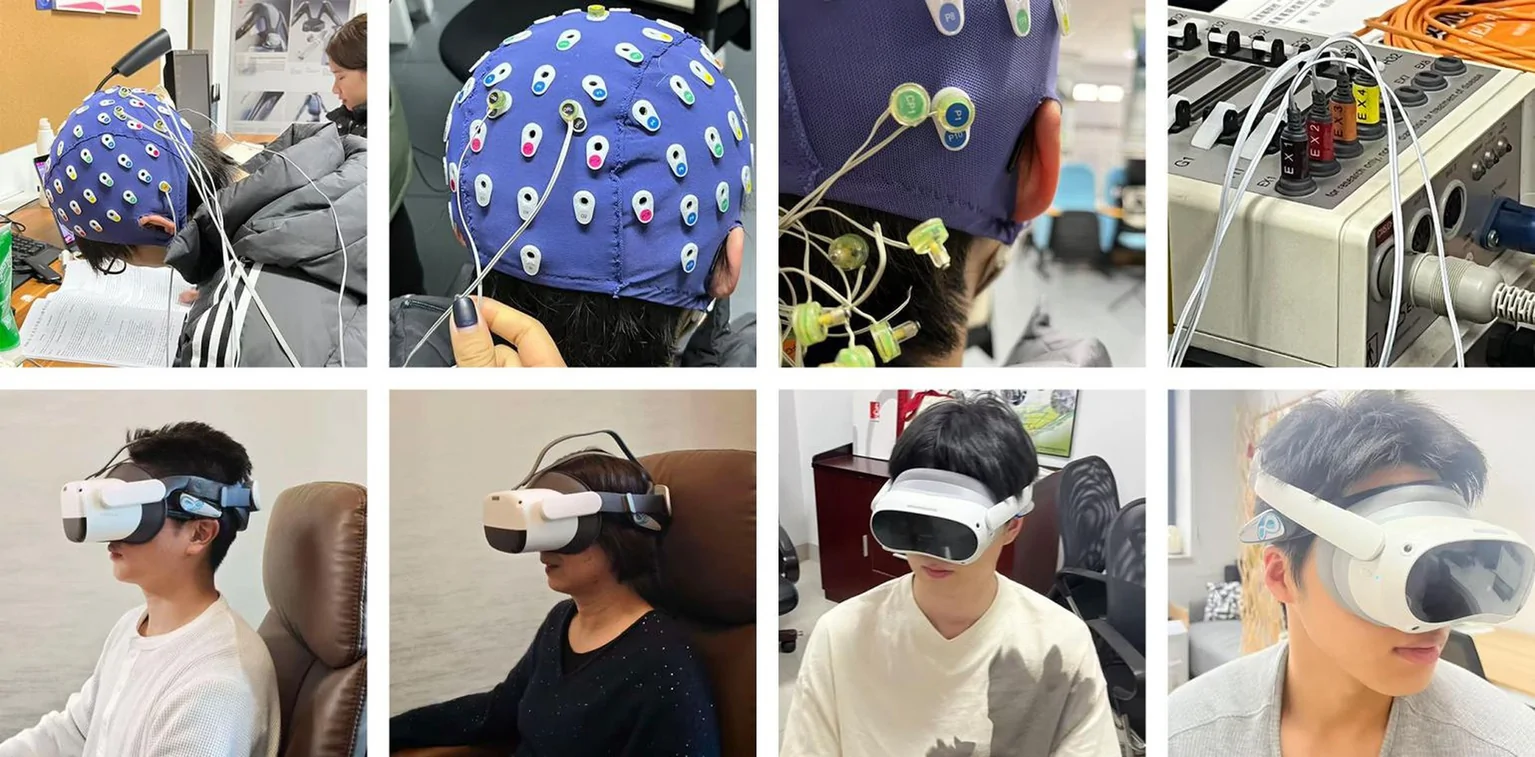
Invited participants undergoing EEG and VR testing.

### 2.3 Tourism context, place meaning, and scene evaluation

The value of tourist streetscapes cannot be reduced to their degree of naturalness. Landmark recognizability can support orientation and memory, heritage features can strengthen sense of place and cultural appeal, and local distinctiveness can differentiate a destination from visually homogenized urban environments (34–37). These attributes may coexist with relatively high levels of visual information: the same density of detail may be experienced as clutter in a transport corridor but as meaningful environmental information in a historic street.

Scene evaluation should therefore be understood as the joint product of visible spatial configuration and observer interpretation. Official heritage status or attraction category can describe the contextual background of a site, but neither demonstrates what meaning a particular photograph communicates to an observer. Conversely, participants’ comfort or restorativeness ratings cannot be reinterpreted as objective properties of a building façade. The analysis retains both levels of evidence while avoiding conflation of contextual classification with immediate psychological evaluation.

Many streetscape attributes may also have nonlinear relationships with experience. Moderate enclosure can provide clear spatial definition, whereas excessive enclosure may restrict views; a certain level of activity may convey vitality, whereas excessive activity may be experienced as crowding; visual complexity may stimulate exploration but may increase information load at high levels. Because the experiment includes only six complete scenes, the present data are insufficient to estimate dose–response curves for these characteristics. These possible mechanisms are used only to interpret scene heterogeneity and motivate future research, not to infer the causal effect of any single feature from the current scene comparisons.

Given this limitation, the complete scene is consistently treated as the experimental unit of comparison. Greenery, sky, openness, hardscape, façade characteristics, heritage status, and color composition vary jointly within the same photograph. The study asks whether the six scenes produce different restoration-related responses and whether different modalities show partially convergent scene patterns, rather than attributing the observed effects to any single background indicator.

### 2.4 Multimodal response mechanisms and measurement boundaries

The value of multimodal measurement lies in observing the same scene across different levels of response. Perceived restorativeness is a subjective evaluation; eye tracking records how attention is allocated but does not directly represent liking (52, 53); EEG provides temporally sensitive spectral information whose interpretation depends on referencing, artifact handling, and preprocessing procedures (54, 55); EDA primarily reflects sympathetic arousal and does not directly encode emotional valence (56); and HRV measures such as RMSSD capture short-term cardiac variability but are likewise influenced by signal quality, beat correction, respiration, and analysis-window length (57–59).

Accordingly, multimodal convergence in this study refers to mutually supportive scene-level patterns across different indicators. If perceived restorativeness, natural-AOI fixation, EEG, EDA, and HRV simultaneously distinguish particular scenes, the combined evidence strengthens interpretation of scene differences; however, the modalities are still allowed to show different rankings or null effects. A scene may, for example, elicit more dispersed visual search without being more restorative, while subjective restorativeness may change even when sustained-attention accuracy remains stable.

Because each participant contributes multiple scene exposures and multiple types of sensor data, the statistical analysis must explicitly account for within-participant dependence. Eye-tracking samples, EEG epochs, and SART events are repeated observations nested within participants and cannot be treated as independent samples. Missingness is also typically modality specific: failure of the eye-tracking record in one trial does not imply that the EEG or EDA record from the same trial is invalid. Repeated-measures models are therefore specified at the participant level, and valid sample sizes and quality-control results are reported separately for each modality.

The interpretability of multimodal research also depends on complete reporting of acquisition and preprocessing information. Reporting guidelines for eye tracking, EEG, EDA, and HRV recommend specification of devices, sampling rates, calibration or referencing procedures, preprocessing, artifact handling, and exclusion criteria because these settings directly affect the meaning of derived measures (53, 55, 56, 59). This study reports only parameters that can be independently verified from the research archive; unverifiable information is retained as metadata to be completed from the original SOPs and device records before submission rather than being replaced with assumed defaults.

### 2.5 Integrated framework and empirical expectations

Based on the theoretical and measurement logic outlined above, this study adopts a sequential design. First, an urban-scale environmental sample of 66 tourism-related sites is established and 11 objective indicators are calculated. Second, PCA is used to summarize the main environmental gradients and K-means is applied to identify six environmental archetypes. Third, without reference to participant outcomes, six permission-cleared field-photography candidate locations are matched one-to-one with the six environmental archetypes. Finally, participants’ subjective, cognitive, eye-tracking, and psychophysiological responses after viewing the six fixed scenes are compared. ART is used to interpret perceived restorativeness, SRT to interpret post-stress neural and autonomic changes, eye tracking to characterize attentional allocation, and the sustained-attention task to assess short-term cognitive performance.

After objective scene screening was completed, three scene-level empirical hypotheses were specified. H1: Perceived restorativeness differs among the six complete scenes after accounting for repeated observations and presentation order. H2: The proportion of fixations on natural AOIs and gaze entropy differ among the six complete scenes, although neither is interpreted directly as preference or as evidence that any single visible feature caused the difference. H3: Changes from the stress phase to the scene phase in EEG alpha-minus-theta, mean EDA SCL, and HRV RMSSD differ among the six complete scenes. These hypotheses concern multivariate scene configurations rather than dose–response effects of greenery, openness, legibility, or any other isolated characteristic. PCA and K-means are used only for upstream scene sampling and do not enter hypothesis testing of the human-response outcomes; cognitive accuracy and cross-modal response profiles are complementary analyses (Table 2).

**Table 2:** Scene-level empirical hypotheses and evidential boundaries.

Item	Empirical expectation	Primary analysis	Interpretive scope
H1	Perceived restoration differs among the six fixed scenes after accounting for repeated observations and presentation order.	Gaussian participant-random-intercept model; omnibus scene test	Scene contrast; not isolated-feature causation
H2	Natural-AOI fixation share and gaze entropy differ among the six fixed scenes.	Endpoint-specific participant-random-intercept models; multiplicity control within the seven-endpoint primary family	Visual-attention allocation; not preference or liking
H3	Stress-to-scene change differs among scenes in EEG alpha-minus-theta, EDA mean-SCL and HRV RMSSD.	Endpoint-specific participant-random-intercept models; BH-FDR across the seven primary tests	Modality-specific psychophysiological recovery; no biological equivalence assumed
Boundary A	The eleven objective indicators are reduced by PCA and grouped by k = 6 K-means before participant outcomes are analyzed.	PCA (five retained PCs) + K-means + one-to-one centroid matching	Sampling/typology bridge only; no feature-level causal attribution
Boundary B	Sustained-attention accuracy and cross-modal profiles are complementary analyses rather than post-hoc replacements for H1–H3.	Event-level clustered model; standardized descriptive profiles; complete-case sensitivity	No universal multimodal quality score

## 3 Materials and methods

### 3.1 Study design and inferential architecture

The study workflow comprised five sequential stages (Figure 1). In Stage 1, 66 tourism-related analytical sites were identified from official sources. In Stage 2, four fixed-direction streetscape views that passed quality control were retained for each site (264 images in total) and combined with GIS information for the surrounding 250-m neighborhood to form a 66 × 11 site-level objective matrix. In Stage 3, the 11 indicators were standardized, PCA was performed, and candidate K-means solutions were examined; k = 6 was retained as a pragmatic six-group sampling solution because the experimental protocol had a pre-specified capacity of six fixed scenes, not because six groups represented a uniquely optimal natural typology. In Stage 4, six permission-cleared field-photography candidate locations were matched to the six k = 6 centroids. In Stage 5, participants viewed standardized static two-dimensional photographs while EEG, eye tracking, EDA, and HRV were recorded, followed by subjective ratings and a sustained-attention task. The analysis compared scene-level outcomes and did not include path analysis, mediation analysis, or structural equation modeling.

This sequence was designed to ensure that scene selection remained independent of the human experimental outcomes. Restorativeness ratings, eye-tracking measures, and physiological responses could not retrospectively alter the site framework, environmental indicators, PCA solution, or clustering results. Clustering was used to achieve coverage of the major dimensions of environmental variation rather than to interpret the six scenes as six mutually independent single-factor conditions. Accordingly, the six complete visual scenes remained the primary objects of comparison in the experimental stage.

### 3.2 Study area, analytical framework, and multi-source urban data

The study area comprised the high-density urban core surrounding Victoria Harbour, covering 22.312 km^2^ across the northern shore of Hong Kong Island and the southern shore of Kowloon and encompassing major tourism, heritage, museum, commercial, and transport-approach spaces (Figure 2). This boundary was defined specifically for the spatial analysis and does not correspond to an administrative unit. The area was selected because, within a continuous high-density urban system, it contains substantial variation in built intensity, street morphology, attraction type, public-space form, and visible natural content.

Candidate sites were drawn from three categories of official open databases: declared monuments with mapped locations, museums, and major attractions (Figure 4). Official English names, source identifiers, coordinates, and geometric information were standardized, and duplicate records were checked before the analytical sample was finalized (61). A total of 66 sites were retained, comprising 48 declared monuments, 11 museums, and 7 major attractions (Figure 4).

**Figure 4:**
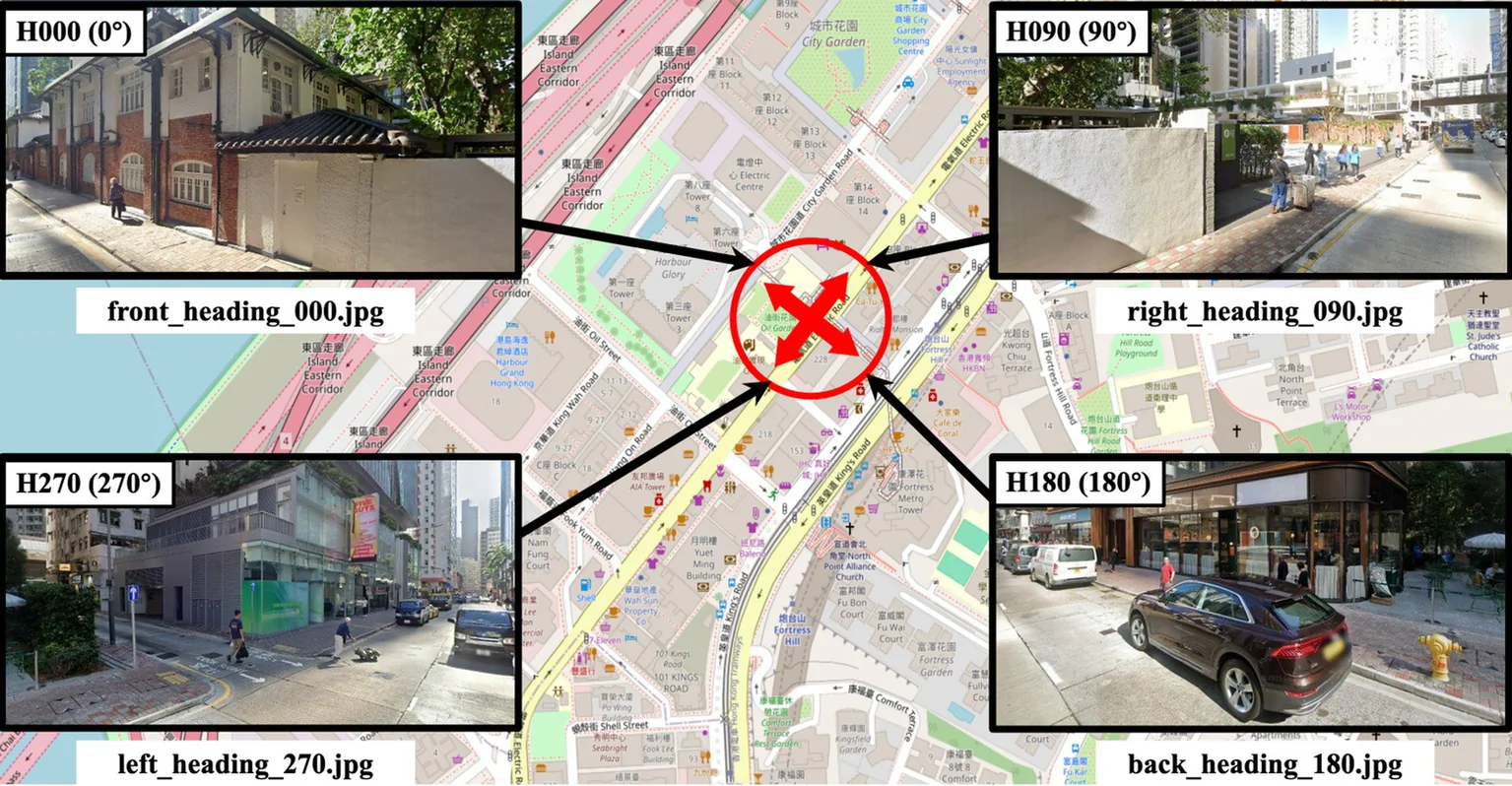
Route-validated four-view sampling geometry for street-level visual exposure.

For each site, Hong Kong 1980 Grid coordinates (EPSG:2326) and source information were retained and linked to the GIS context and four fixed-direction streetscape observations, yielding a complete sample of 66 sites × 4 directions (66). Table 3 summarizes the different data layers and their roles in the study.

**Table 3:** Multisource data layers and their analytical roles.

Data layer	Unit / extent	Key variables	Role in the present study
Official site frame	66 sites	Name, source family, HK80 coordinates, boundary relation	Defines the objective analytical frame
GIS morphology	100/250/500 m buffers	Building coverage, height proxies, road length/density, mapped Harbour Limit distance	Characterizes surrounding urban form
Route-validated imagery	264 views; four headings/site	Image files, view identifiers, provenance and quality fields	Provides pedestrian-level objective visual exposure
Deterministic image proxies	View and site mean	Green/blue/sky color share, entropy, saliency, image-quality audit	Transparent baseline visual indicators; not semantic segmentation
Research-team photographs	Six fixed scenes	Standardized files, hashes and permissions	Controlled experimental stimuli
Participant/trial archive	108 focal exposures from 46 of 48 registered adults	Participant, session, trial, scene, order, subjective and cognitive outcomes	Defines repeated-observation experimental cohort
Multimodal recordings	Trial/stage specific	Gaze, EEG, EDA, HRV and modality QC	Tests attentional, neural and autonomic scene responses
PCA/K-means selection bridge	66 sites → 5 retained PCs → 6 centroids	PCA scores, cluster labels, centroids, candidate-to-centroid distances	Defines six environmental archetypes and links field-photographed candidates before outcome analysis

### 3.3 Objective urban morphology, streetscape indicators, and PCA/K-means scene screening

Five GIS-based contextual indicators were calculated within a 250-m neighbourhood around each site. The 250-m radius was used to characterize the immediate approach environment surrounding an attraction while preserving sufficient between-site variation within the high-density Victoria Harbour core; 100-m and 500-m radii were used only for scale-sensitivity analyses and were not entered into the 11-variable PCA. Building footprints and road centrelines were clipped to circular 250-m buffers in EPSG:2326. The five retained indicators were building coverage ratio (BCR), a built-volume-density proxy (BVD), mean building-height proxy, road density, and shortest planar distance to the statutory Harbour Limit.

$$\mathrm{BC}R_{s}=\frac{\sum A_{\mathrm{sk}}}{A_{250}}$$ (1)

$$\mathrm{BV}D_{s}=\frac{\sum (A_{\mathrm{sk}}\times h_{k})}{A_{250}}$$ (2)

$$RD_{s}=\frac{L_{s}}{\left(\frac{A_{250}}{10,000}\right)}$$ (3)

In Equations 1–3, A₂₅₀ = π(250 m)^2^; Aₛk is the clipped area of building footprint k within the 250-m buffer of site s; hₖ is the source-derived height proxy for that building; and Lₛ is the total length of clipped road centrelines within the buffer. BCR is dimensionless, BVD and mean building height are expressed in meters, and road density is expressed in m ha^−1^. Four quality-checked streetscape images were retained for each site at the nominal headings H000, H090, H180, and H270. Only sites for which all four directions passed quality control were included in the subsequent PCA (65).

The 11 indicators used in PCA were specified in advance and comprised five GIS-based contextual indicators and six streetscape-image proxies. The GIS indicators were: (1) 250-m BCR; (2) 250-m BVD proxy; (3) 250-m mean building-height proxy; (4) 250-m road density; and (5) distance to the statutory Harbour Limit. The image indicators were: (6) green-color view proxy; (7) blue-color view proxy; (8) upper-image sky-color proxy; (9) natural-color saliency proxy; (10) luminance Shannon entropy; and (11) technical image-quality proxy. Raw road-centreline length was not included as an additional PCA variable because, within a fixed 250-m buffer, it is linearly redundant with road density. Table 4 reports the calculation method, unit, site-level aggregation procedure, and interpretive scope of each indicator.

**Table 4:** Objective indicators, calculations and interpretive limits.

Domain	Indicator	Calculation/Site aggregation
GIS morphology	Building coverage ratio (BCR), 250 m	Σ clipped footprint area / π250^2^; dimensionless
GIS morphology	Built-volume-density proxy (BVD), 250 m	Σ(clipped footprint area × source height) / π250^2^; m
GIS morphology	Mean building-height proxy, 250 m	Mean retained source-height proxy for buildings intersecting 250-m buffer; m
GIS/network	Road density, 250 m	Total clipped road-centreline length / buffer hectares; m ha^−1^
Harbour context	Distance to mapped Harbour Limit	Shortest planar EPSG:2326 distance from site point to legal Harbour Limit; m
Street-view color	Green-color view proxy	Fixed v0 green-color pixel share per view; arithmetic mean of four headings
Street-view color	Blue-color view proxy	Fixed v0 blue-color pixel share per view; arithmetic mean of four headings
Street-view color	Upper-field sky-color proxy	Fixed sky-color rule in designated upper field; mean of four headings
Street-view structure	Natural-color saliency proxy	Archived v0 natural-color saliency response; mean of four headings
Street-view structure	Luminance Shannon entropy	−Σ p log₂p per view; mean of four headings; bits
Technical QA	Image-quality proxy	Bounded v0 brightness/contrast capture-quality audit; mean of four headings

The PCA and clustering results were used to identify six representative environmental positions from which the corresponding four-direction streetscape images could be examined. Image indicators for these six sites were calculated using the same archived color–texture processing rules applied to all other sites to ensure comparability. The green, blue, and sky proxies represent the proportions of pixels satisfying their respective color rules; natural-color saliency represents saliency responses associated with naturally colored regions; luminance entropy was calculated from the luminance histogram; and the technical image-quality proxy was used to identify conspicuous luminance or contrast abnormalities. Values from the four directions were then arithmetically averaged to obtain site-level measures (Figure 5). Figure 6 presents the five GIS indicators and six streetscape-image proxy indicators.

**Figure 5:**
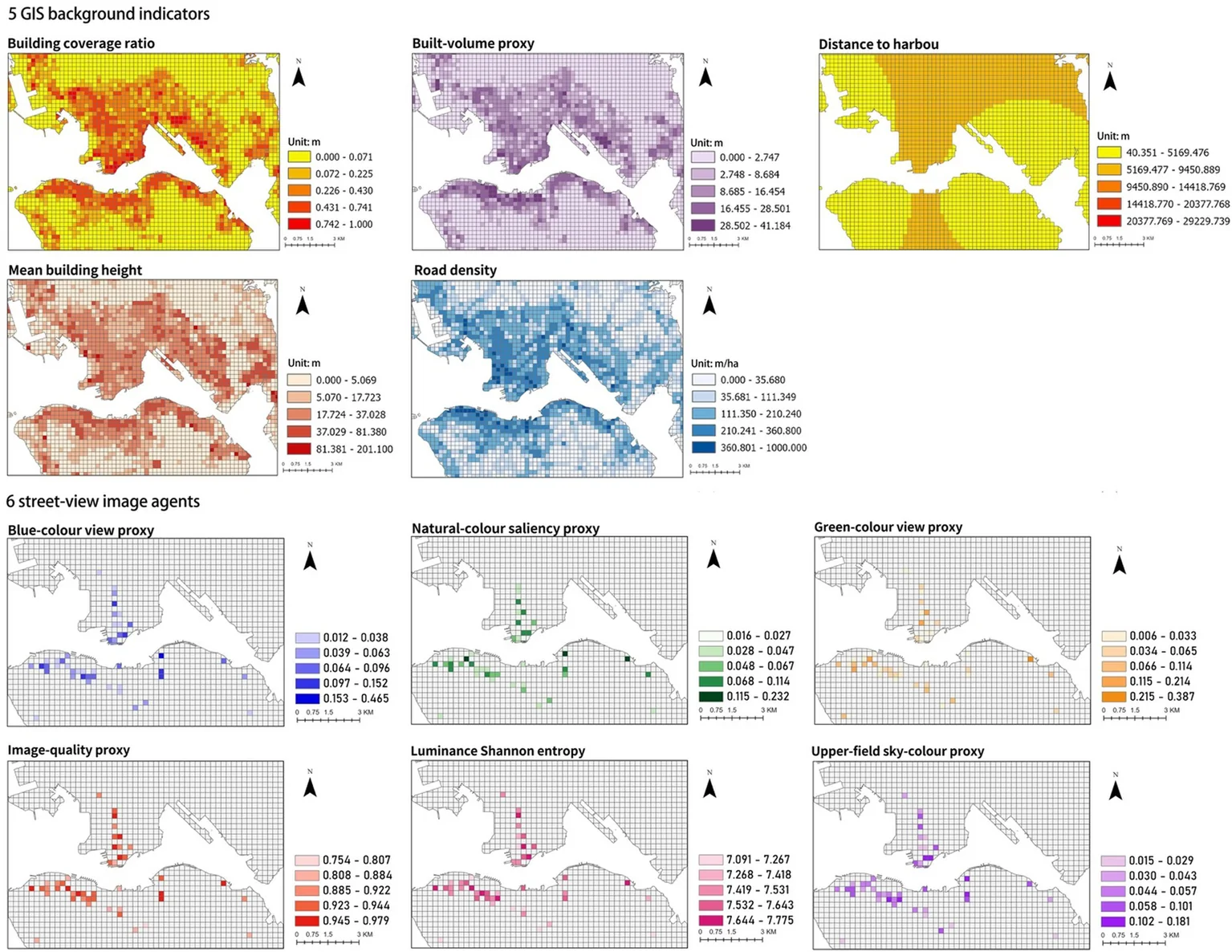
Spatial distributions of retained GIS indicators at 250 m.

**Figure 6:**
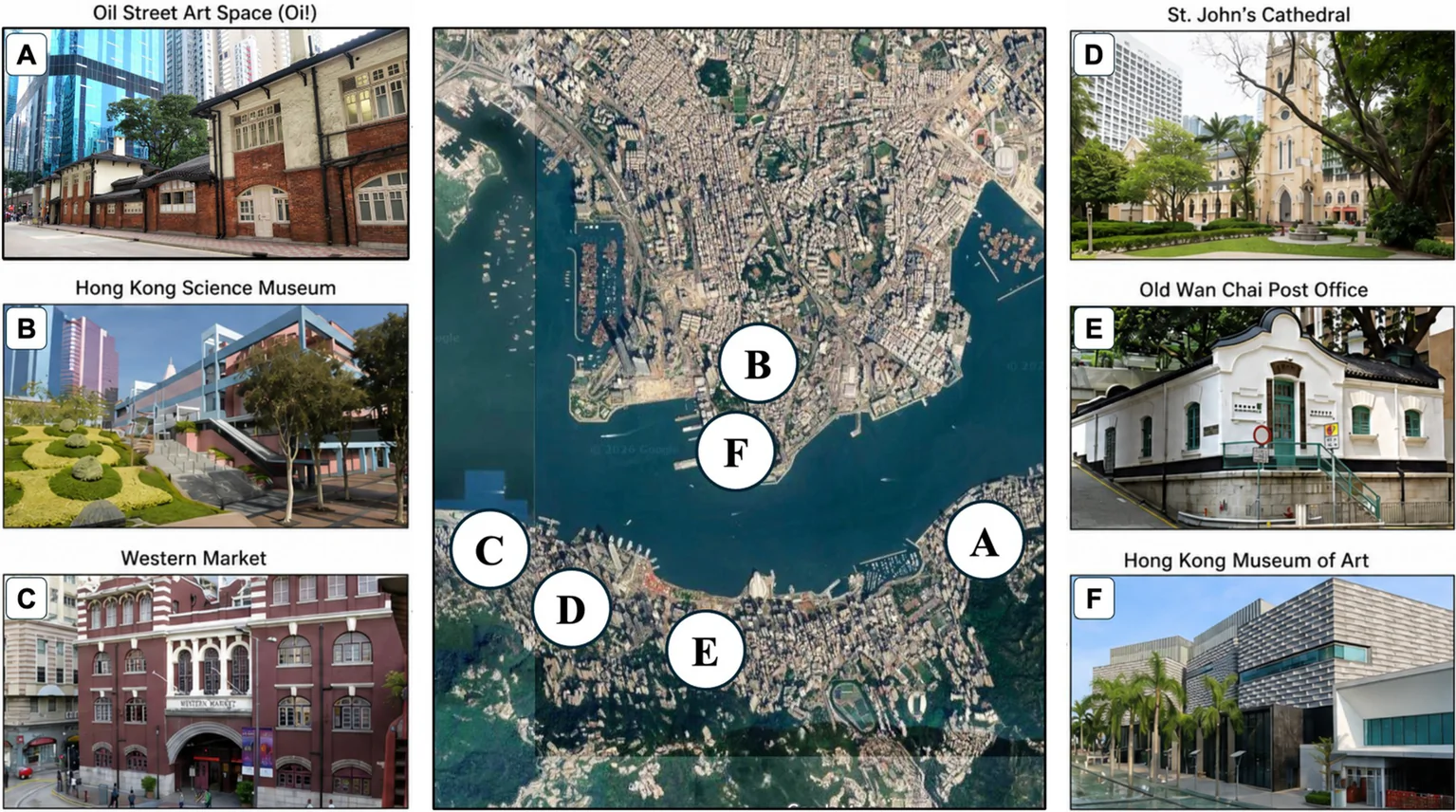
Six experimental sites matched to environmental cluster centroids.

The indicators illustrated in Figure 7 are interpreted as reproducible color and image-structure proxies rather than as substitutes for semantic segmentation. To aggregate view-level information to the site level, the value of any image indicator q was averaged with equal weight across the four directions at the same site according to Equation 4. Each site therefore contributed a single row to PCA and clustering, preventing unequal numbers of images from introducing additional weighting. The original values for all four directions were retained for data auditing and assessment of directional variation.

$${\overset{`}{q}}_{s}=\frac{1}{4}\;\sum_{i=1}^{4}q_{\mathrm{si}}$$ (4)

$$H_{i}=-\sum_{b}\;p_{\mathrm{ib}}\;\log_{2}(p_{\mathrm{ib}})$$ (5)

$$z_{\mathrm{sj}}=\frac{\left(x_{\mathrm{sj}}-{\bar{x}}_{j}\right)}{s_{j}}$$ (6)

**Figure 7:**
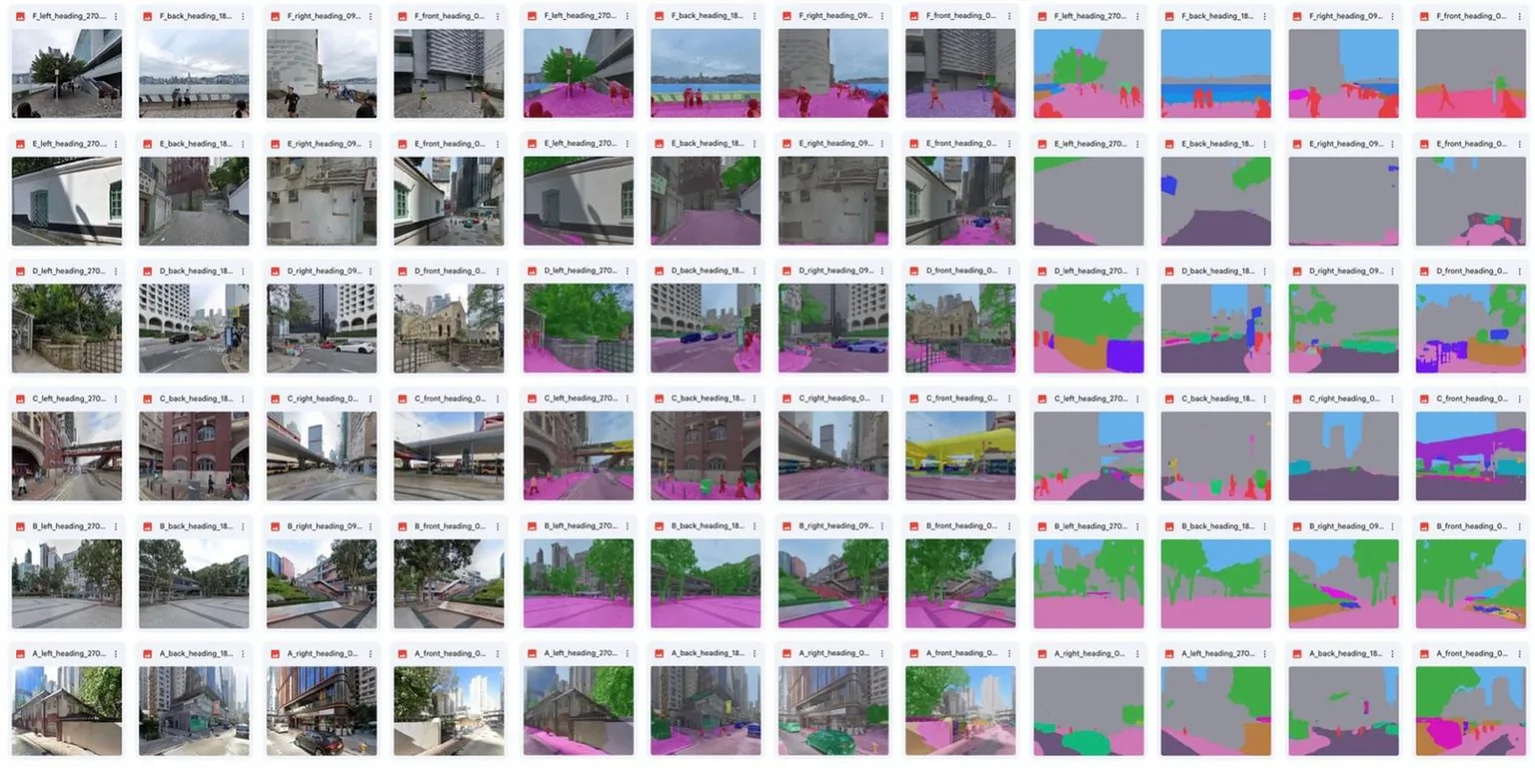
Segmentation of source photographs for two-dimensional visual-stimulus scene database construction.

Equation 5 defines luminance Shannon entropy for a single image, where pᵢᵦ denotes the proportion of pixels in luminance bin b. Equation 6 defines site-level standardization, where xₛⱼ is indicator j for site s and x̄ⱼ and sⱼ are, respectively, the mean and standard deviation of that indicator across the 66 sites. Because the 11 indicators comprise proportions, distances, densities, bits, and bounded scores with different units and numerical ranges, applying PCA directly would assign disproportionate weight to variables with larger numerical scales. All indicators were therefore converted to z-scores, yielding a dimensionless 66 × 11 matrix for PCA.

The purpose of PCA was to summarize the 11 correlated environmental indicators into a small number of dominant environmental gradients rather than to produce a single streetscape quality score. PCA was applied to the standardized 66 × 11 matrix using full singular value decomposition (SVD) without whitening. PC1, PC2, and PC3 explained 25.4%, 21.4%, and 14.3% of the variance, respectively; the first five principal components together explained 80.14% and were retained for clustering. Candidate K-means solutions from k = 2–8 were compared as an internal sensitivity check (Supplementary Table A2). Although k = 6 is not the only optimal solution: for example, k = 4 produced a little higher silhouette coefficient than k = 6 (0.254 vs. 0.226), while k = 6 had a lower Davies–Bouldin index than k = 4 or k = 5 (1.092 vs. 1.246 and 1.303) but included a singleton cluster. After comprehensively considering various experimental results, k = 6 was retained as a sampling solution. The resulting groups are therefore treated as sampling strata/archetypal positions for this study. The six field-photography candidates were then matched one-to-one with the six centroids (Supplementary Figures A1–A5).

### 3.4 Cluster-guided experimental scenes and participant cohort

Centroid matching yielded the six fixed experimental scenes shown in Figure 7 and Table 5. In A–F order, these were Oil Street Art Space (Oi!), Hong Kong Science Museum, Western Market, St. John’s Cathedral, Old Wan Chai Post Office, and Hong Kong Museum of Art. The six locations differed substantially in built intensity, harbour proximity, visible color-proxy composition, heritage-street interface, and public-space configuration, thereby spanning distinct positions in the retained PCA space.

**Table 5:** Six-scene experimental stimulus set.

Scene	Site	Location	Matched PCA–K-means archetype	PC1/PC2
A	0067	Oil Street Art Space (Oi!)	A (*n* = 1)	5.198/1.529
B	0063	Hong Kong Science Museum	B (*n* = 13)	2.019/0.560
C	0048	Western Market	C (*n* = 21)	0.542 / -2.732
D	0038	St. John’s Cathedral	D (*n* = 6)	1.800/0.041
E	0036	Old Wan Chai Post Office	E (*n* = 16)	−0.603 / -2.453
F	0061	Hong Kong Museum of Art	F (*n* = 9)	−2.637/0.288

The six source photographs were captured on site by members of the research team or obtained from permission-cleared open sources and standardized as static two-dimensional experimental stimuli. Segment Anything Model (SAM) outputs were used to support semantic annotation of the experimental stimulus set and the definition/checking of scene-level AOIs (Figure 3; Supplementary Figures A6, A7).

The participant registry included 48 eligible adults who provided informed consent (Figure 8). Exact matching of the verified master trial table to the six focal scene identifiers showed that 46 participants contributed at least one focal-scene exposure, yielding 108 unique participant-by-scene records; two registered participants completed their study sessions but did not contribute a trial from the six focal-scene subset analyzed here. Among the 46 focal-scene contributors, 10 contributed one focal exposure, 14 contributed two, 18 contributed three, and 4 contributed four. The resulting focal dataset therefore had an incomplete repeated-measures structure (Table 6).

**Figure 8:**
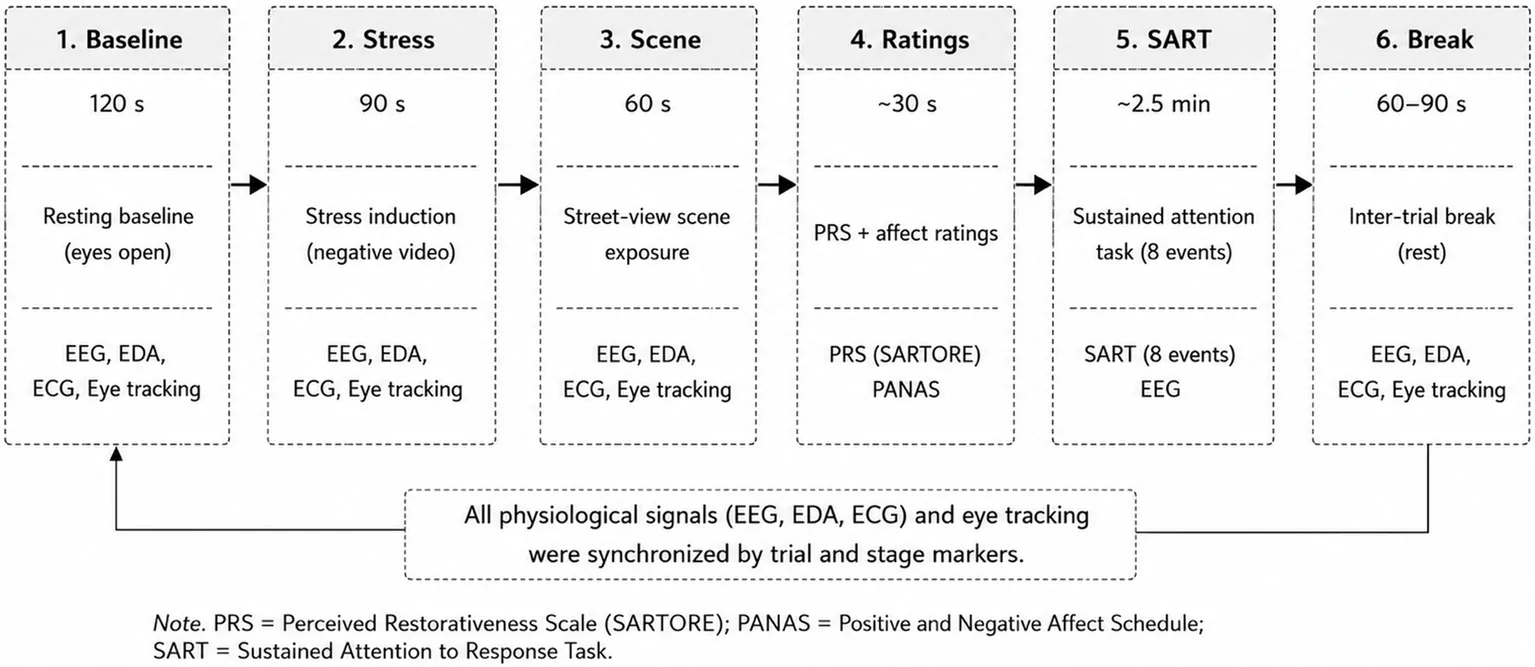
Experimental trial sequence and multimodal assessment stages.

**Table 6:** Recorded participant, subjective, cognitive, trial and quality-control variables.

Block	Recorded variables	Scale/Unit
Participant context	Age, sex, sleep, caffeine/exercise context	Mixed
Restoration	Being away, fascination, coherence/extent, compatibility, PRS total	1–7
Affect	Stress, calmness, tension, positive affect	1–7
Cognitive	SART response, correct, RT, omission, commission	8 events/trial
Trial structure	Participant, session, trial, scene, order, stage	Identifiers/time
Quality control	Subjective, cognitive, eye, EEG, EDA, HRV validity	Boolean
Cohort rule	Six focal scene IDs intersected with verified master trial IDs	Exact match

The focal-scene analysis set was derived by exact matching of the verified master trial registry to the six scene identifiers. No subjective, eye-tracking, or physiological outcome was used in determining scene inclusion.

### 3.5 Multimodal experimental procedure, outcome construction, and quality control

The experimental procedure followed a standardized sequence: baseline → mild stress induction → post-stress measurement → static scene exposure with simultaneous EEG, eye-tracking, EDA, and HRV recording → subjective ratings → sustained-attention task → rest/next trial (Figure 9). The approved implementation treated the stress induction as low-risk and interruptible. Participants could request a pause or discontinue without being required to complete additional trials; experimenters were instructed to monitor fatigue/discomfort and to use the planned break/withdrawal procedure rather than prioritize data completion. All 48 session logs were marked as completed, and no participant-level withdrawal was recorded. These procedures do not eliminate fatigue or habituation as potential within-session influences, which were therefore examined explicitly in sensitivity analyses.

**Figure 9:**
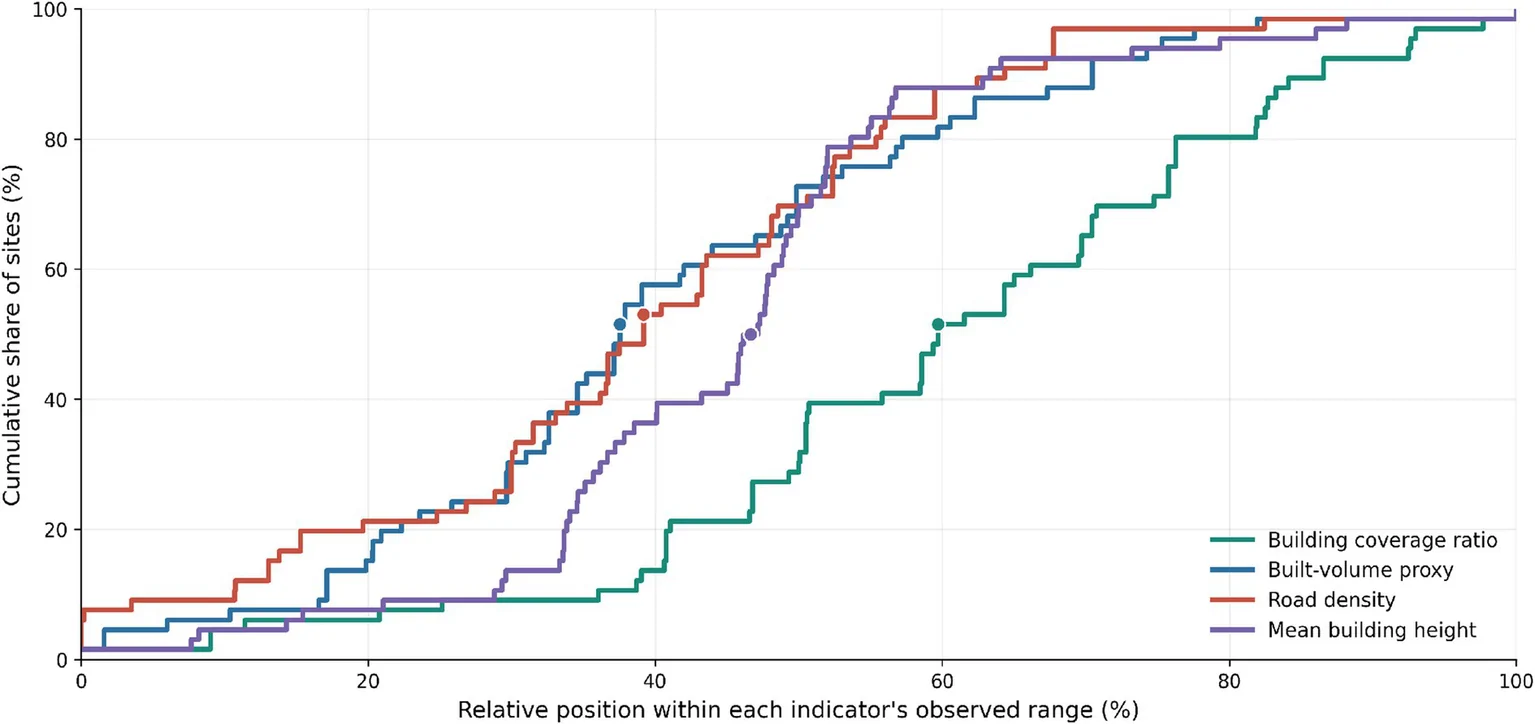
Empirical cumulative distributions of four built-morphology indicators.

The primary subjective outcome, the Perceived Restorativeness Score (PRS), was calculated as the mean of ratings for four dimensions: being away, fascination, coherence/extent, and compatibility. Stress, calmness, tension, and positive affect were treated as secondary subjective outcomes. Short-term cognitive performance was assessed using a sustained-attention task. After each scene exposure, participants completed an eight-event SART block comprising seven go events and one no-go event; the 108 valid scene exposures therefore yielded 864 event-level observations. Response correctness was used to analyze accuracy, while reaction time for correct, non-omission responses was treated as a secondary cognitive outcome.

Eye-tracking outputs included tracking ratio, number of valid gaze samples, fixation count and duration, saccade amplitude, gaze entropy, and fixation counts within AOIs corresponding to vegetation, water, sky, buildings, traffic, commercial/signage elements, and people. The proportion of fixations on natural AOIs was defined as the combined fixation count for vegetation, water, and sky divided by the total fixation count across all AOIs. The experimental protocol required eye-tracker calibration and validation before scene exposure and documentation of missing samples and tracking ratio.

The EEG trial table retained valid duration, number of bad channels, artifact proportion, and number of usable epochs for the baseline, stress, and scene phases. The archived feature table contained log-band power (log μV^2^) for theta, alpha, and beta bands across frontal, central, parietal, occipital, and temporal regions. The primary neural outcome was defined as the change from the stress phase to the scene phase in log alpha-minus-theta averaged across the five brain regions.

For EDA, mean skin conductance level (SCL) was used as the primary measure of sympathetic arousal, with SCR count and phasic-related measures retained as secondary outcomes. HRV was represented primarily by RMSSD calculated from archived PPG/inter-beat-interval signals, with SDNN, HF-related indices, and mean heart rate retained as secondary measures. For both modalities, the principal change score was defined as scene minus stress. Quality control was applied separately by modality: records were excluded from a modality-specific primary analysis when valid-signal coverage fell below 80% or when the predefined artifact rule was exceeded; failure of one sensor did not remove other valid modalities from the same trial (Table 7).

**Table 7:** Primary multimodal endpoints, contrasts and interpretive constraints.

Modality	Primary analysis endpoint	Processing/Contrast	Constraint
Subjective	Perceived restoration total	Mean of four archived restoration fields; post-scene	Appraisal, not objective exposure
Cognitive	SART event accuracy	Participant-clustered binary model	Short task; practice/order retained
Gaze	Natural-AOI fixation share; entropy	Trial QC; seven AOIs	Attention is not preference
EEG	Log alpha-minus-theta change	Five-region mean log(alpha − theta); scene − stress	Archive-defined spectral contrast; preprocessing metadata incomplete
EDA	Mean SCL change	Mean SCL; scene − stress	Arousal is valence ambiguous
HRV	RMSSD change	PPG/IBI-derived RMSSD; scene − stress	Short-window PPG-derived HRV; beat-correction metadata to be verified

Event-marker records indicated that each trial proceeded in the following sequence: 120 s baseline, 90 s mild stress induction, 60 s static scene exposure, 20 s rating, 45 s sustained-attention task, and 30 s inter-trial break. EEG timestamps were used as the reference for cross-device synchronization; relative offsets for eye tracking, EDA, and HRV were +3 ms, −4 ms, and +5 ms, respectively, all within 5 ms. All modalities were linked using participant, session, trial, scene, presentation order, and stage identifiers. The six focal scenes were not evenly distributed over presentation positions 1–10 (Supplementary Table A4); presentation order was therefore included as a covariate in the primary models and was examined more directly through scene-by-order interaction, signal-quality-by-order, and earliest-focal-exposure sensitivity analyses.

### 3.6 Statistical analysis, sensitivity analysis, and reproducibility

Objective environmental analysis first assessed completeness of the 11 site-level indicators and reported the mean, standard deviation, median, interquartile range, and range, while Spearman correlations described monotonic relationships among indicators. The complete 66 × 11 matrix was standardized and subjected to PCA, retaining principal-component axes that together explained 80.14% of the variance. K-means solutions for k = 2–8 were compared as an internal sensitivity check; k = 6 was retained for the pre-specified six-scene sampling capacity rather than declared statistically optimal. Finally, the six field-photography candidate sites were linked to the six k = 6 centroids through one-to-one matching that minimized total centroid distance.

Seven primary outcomes were defined *a priori* for the focal-scene experimental analysis: perceived restorativeness, cognitive accuracy, proportion of fixations on natural AOIs, gaze entropy, change in EEG alpha-minus-theta, change in mean EDA SCL, and change in HRV RMSSD. Ninety-five per cent confidence intervals for scene means were estimated using participant-clustered bootstrap resampling. Continuous outcomes were analyzed with Gaussian participant-random-intercept models, with scene specified as a six-level fixed effect, participant as a random intercept, and presentation order as a covariate. The overall scene effect was assessed using a likelihood-ratio χ^2^ test comparing models with and without the scene term (62). Cognitive accuracy was analyzed at the SART event level using participant-clustered logistic inference. The seven primary *p* values were controlled jointly using BH-FDR (64).

$$\mathrm{PR}S_{\mathrm{ps}}=\frac{\left(BA_{\mathrm{ps}}+FA_{\mathrm{ps}}+CE_{\mathrm{ps}}+CO_{\mathrm{ps}}\right)}{4}$$ (7)

$$\Delta Y_{\mathrm{ps}}=Y_{\mathrm{ps}},\text{scene}-Y_{\mathrm{ps}},\text{stress}$$ (8)

$$y_{\mathrm{ps}}=\beta_{0}+\sum_{j\in \{B,\ldots ,F\}}\beta_{j}I(\text{Scen}e_{s}=j)+\beta_{o}\text{Orde}r_{\mathrm{ps}}+u_{p}+\varepsilon_{\mathrm{ps}}$$ (9)

In Equation 7, PRS denotes perceived restorativeness, and BA, FA, CE, and CO denote ratings of being away, fascination, coherence/extent, and compatibility, respectively (1–7). Equation 8 defines the change in each physiological measure from the stress phase to the scene phase. Equation 9 specifies the mixed model for continuous outcomes, where yₚₛ is the outcome for participant *p* under scene s, βⱼ represents the fixed effect of Scenes B–F relative to the reference scene, βₒ adjusts for presentation order, uₚ is the participant-specific random intercept, and εₚₛ is the residual error (Table 8).

**Table 8:** Experimental cohort, modality-specific denominators, and primary omnibus scene tests.

Endpoint	Trials/People	Omnibus χ^2^	df	*p*	FDR q	Descriptive scene contrast
Perceived restoration	108/46	103.67	5	<0.001	<0.001	A 5.69 highest; C 4.17 lowest
Cognitive accuracy	108/46	5.64	5	0.342	0.342	No adjusted scene effect; A 87.5%
Natural-AOI fixation share	107/46	214.58	5	<0.001	<0.001	A 51.4% highest; C 31.2% lowest
Gaze entropy	107/46	25.01	5	<0.001	<0.001	F 3.39 highest; B 2.95 lowest
EEG alpha-minus-theta change	107/46	69.58	5	<0.001	<0.001	A + 2.68 strongest increase
EDA mean-SCL recovery	107/46	15.93	5	0.007	0.008	Significant scene-specific SCL change
HRV RMSSD recovery	101/45	20.78	5	<0.001	0.001	A + 11.37 ms largest increase

Cognitive accuracy was analyzed at the SART event level as a binary outcome using logistic regression, with scene and presentation order specified as fixed effects and robust sandwich covariance estimated with participant as the clustering unit (60). The overall scene effect was evaluated using a cluster-robust Wald χ^2^ test. Physiological analyses used the scene-minus-stress change scores defined in Equation 8. Because the six experimental scenes were fixed by the study design, scene was treated as a fixed effect; the inferential objective was to compare overall differences among the six complete scenes (Figure 10).

**Figure 10:**
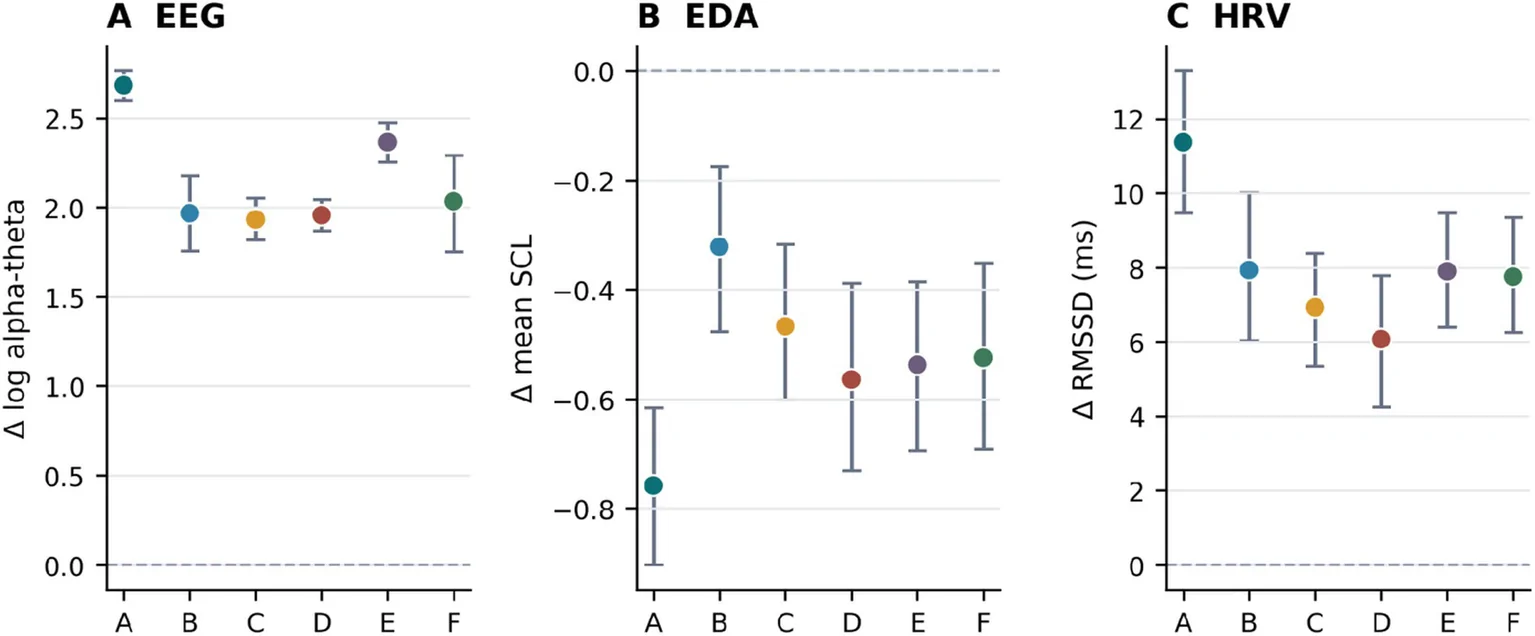
Stress-to-scene EEG, EDA and HRV changes with bootstrap intervals.

For each of the seven pre-specified primary outcomes, one overall scene test was included in the BH-FDR family. Four additional sensitivity checks were conducted specifically in response to the incomplete and unbalanced repeated-measures structure (63). First, participant-clustered models tested scene × presentation-order interaction terms for each primary endpoint. Second, linear order trends were examined for eye-tracking ratio, EEG artifact percentage, EDA valid-signal percentage, and correct-response SART reaction time. Third, an independent-observation sensitivity set retained only the earliest focal-scene exposure contributed by each participant; a literal restriction to presentation order 1 was not used for six-scene comparison because only 10 focal observations occurred at order 1 and Scene F had none. Fourth, modality-specific missingness was tabulated by scene and order, and EEG, EDA, and HRV scene contrasts were re-estimated in the 99-trial complete-case subset and compared with their available-case analyses. These sensitivity tests are reported separately from the seven primary BH-FDR tests (Supplementary Tables A4–A6).

## 4 Results

### 4.1 Cohort resolution, exposure balance, and data quality

Of the 48 registered participants, 46 contributed at least one exposure to one of the six focal scenes, yielding 108 unique participant-by-scene observations. Ten participants contributed one focal exposure, 14 contributed two, 18 contributed three, and 4 contributed four. Two registered participants completed their sessions but did not contribute a trial from the six focal-scene subset. Exposures were unequal across scenes (A–F: 17, 16, 22, 20, 22, and 11) and were unevenly distributed across presentation positions 1–10 (Supplementary Table A4).

The final number of valid focal exposures differed slightly across modalities because quality control was applied independently. PRS and cognitive analyses used 108 trials; eye-tracking and EEG each retained 107; EDA retained 107; and HRV retained 101. A total of 99 trials contained complete records for all seven primary outcomes. Missingness was sparse and modality specific: the single eye-tracking invalid trial occurred in Scene F at order 2; the single EEG invalid trial occurred in Scene D at order 2; the single EDA invalid trial occurred in Scene E at order 4; and the seven HRV invalid trials occurred in Scenes C (*n* = 2), D (*n* = 2), E (*n* = 2), and F (*n* = 1) at orders 1, 2, 3, 5, 5, 7, and 9. Thus, missing records were not concentrated only in late trials, although the sparse number of failures limits formal missingness modeling (Supplementary Table A4).

Correct-response SART reaction time shortened modestly with order, consistent with a small practice/speeding effect. The scene-by-order sensitivity models were endpoint specific: interactions were not significant for PRS, natural-AOI fixation share, gaze entropy, or SART accuracy, but were significant for EEG, EDA, and HRV. These results motivated cautious interpretation of physiological contrasts and additional earliest-focal and complete-case analyses (Supplementary Tables A5, A6).

### 4.2 Environmental heterogeneity across 66 sites and archetypal positioning of the six experimental scenes

The 66 official-source sites exhibited substantial heterogeneity in both urban morphology and pedestrian-level visual environment (Table 9). Within 250 m, building coverage ranged from 0 to 0.719, road density from 0 to 602.6 m ha^−1^, and distance to the Harbour Limit from 475.4 to 6573.5 m; the maximum values of the green- and blue-color proxy indicators were 0.387 and 0.465, respectively (Figures 6, 11). The analytical framework therefore captured high-intensity central urban areas, relatively open public spaces, harbour-proximate contexts, and sites with comparatively high visible natural content, providing a multidimensional environmental sample.

**Table 9:** Objective environmental indicator descriptive statistics across 66 sites.

Indicator	Unit	Mean	SD	Median	IQR	Range
Building coverage ratio, 250 m	Ratio	0.428	0.159	0.429	0.336–0.545	0.000–0.719
Built-volume proxy, 250 m	M	14.892	7.649	13.802	10.899–19.380	0.000–36.774
Mean building-height proxy, 250 m	M	29.6	7.7	30.3	25.1–32.6	9.8–53.8
Road density, 250 m	m ha^−1^	232.7	126.7	236.0	175.3–315.5	0.0–602.6
Distance to mapped Harbour Limit	M	4382.2	1580.9	4302.6	3427.6–5970.0	475.4–6573.5
Green color proxy	Share	0.060	0.066	0.035	0.022–0.072	0.004–0.387
Blue color proxy	Share	0.065	0.061	0.053	0.032–0.079	0.012–0.465
Sky color proxy	Share	0.058	0.036	0.047	0.035–0.072	0.015–0.181
Natural-color saliency	Score	0.061	0.038	0.050	0.037–0.075	0.010–0.232
Luminance entropy	Bits	7.497	0.188	7.507	7.358–7.623	7.091–7.834
Technical image-quality proxy	score	0.916	0.043	0.928	0.884–0.949	0.754–0.979

**Figure 11:**
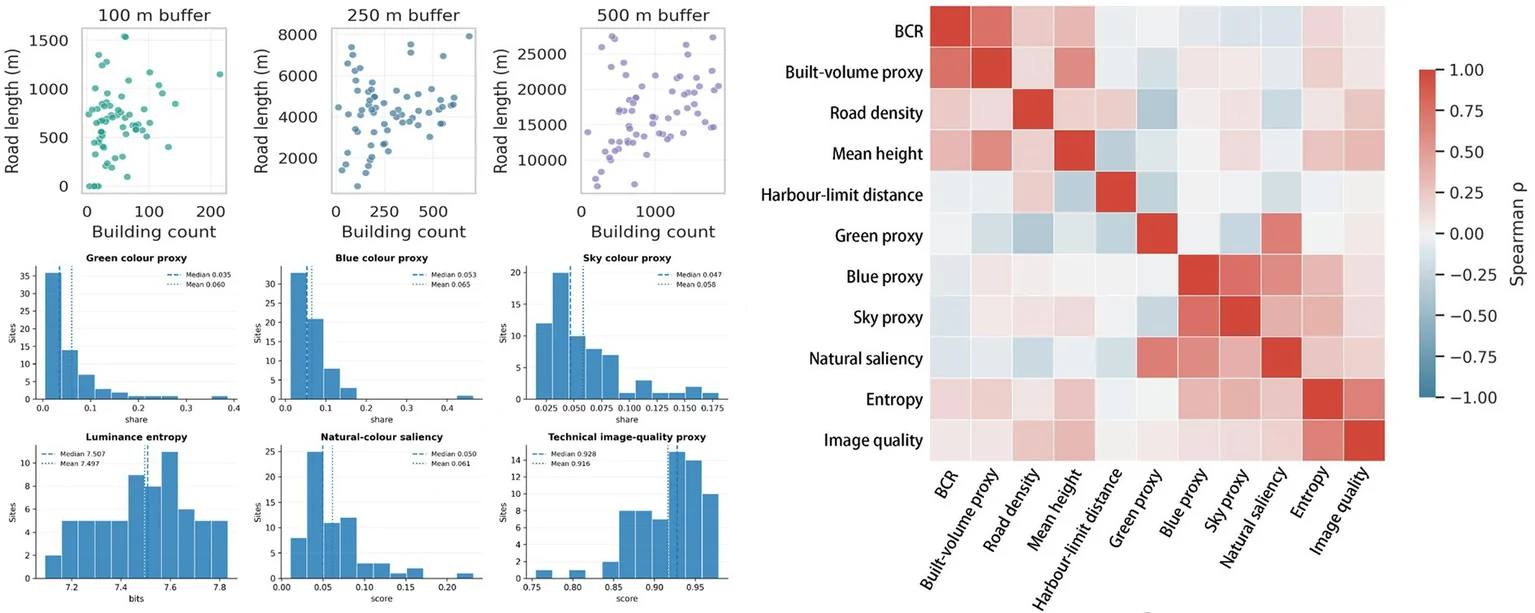
Scale dependence, distributions, and interrelationships of environmental indicators.

Comparison of 100-, 250-, and 500-m buffers showed systematic scale-related changes in building count, building-footprint area, building height, and road-centreline length, while relative differences among sites remained evident (Figure 12). This supports the use of 250 m as the primary scale for the attraction approach environment: it captures the immediate spatial context surrounding a destination without over-smoothing local variation to the extent observed at larger scales. The 100- and 500-m buffers were used only to test whether the findings depended on a single spatial scale.

**Figure 12:**
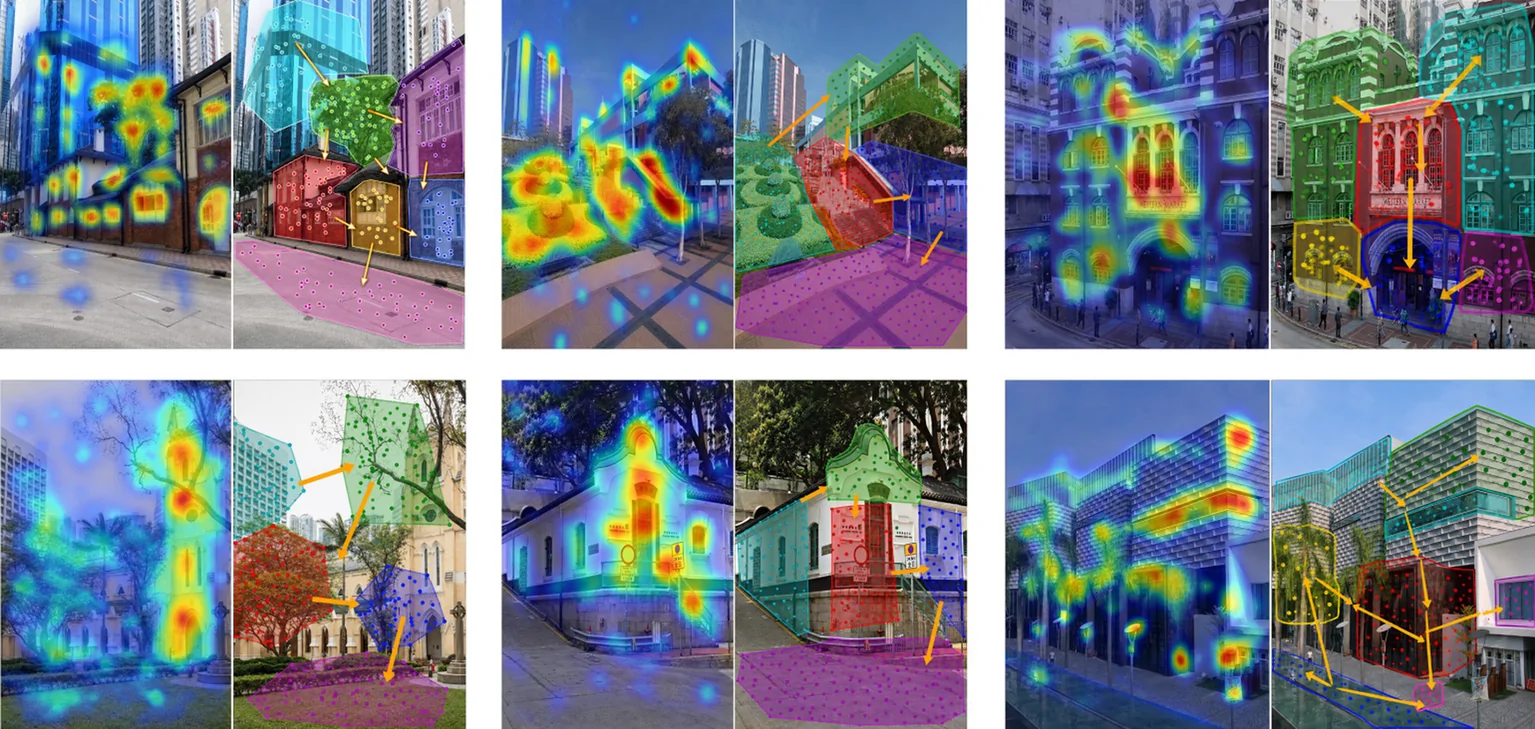
Eye-tracking Gaze heatmap.

Marked differences were also evident at the streetscape level. The green-color proxy was strongly right-skewed (mean 0.060, median 0.035, range 0.004–0.387), indicating that most high-density sites had relatively little visible green content while a small number formed pronounced high-value cases. The blue proxy was also right-skewed (mean 0.065, median 0.053, range 0.012–0.465). In contrast, luminance entropy (mean 7.497 bits, SD 0.188) and the technical image-quality proxy (mean 0.916, SD 0.043) were more tightly distributed. Figure 12 therefore shows that the principal differences among the 66 sites arose not only from built form but also from uneven exposure to natural and sky-related colors at pedestrian eye level.

Several strong correlations were observed among the proxy indicators (Figure 12). The highest correlation was between the blue and sky proxies (ρ = 0.762), reflecting potential overlap in the blue-color information captured by the rules. Natural-color saliency correlated with the green proxy (ρ = 0.665), while luminance entropy correlated with the technical image-quality proxy (ρ = 0.658). These relationships support the use of PCA for dimensionality reduction.

PCA showed that PC1, PC2, and PC3 explained 25.4%, 21.4%, and 14.3% of the variance, respectively, while the first five principal components together explained 80.14%. From the result we can see, k = 6 is not the only optimal solution, in particular, k = 4 had a little higher silhouette coefficient than k = 6 (0.254 vs. 0.226), whereas k = 6 had a lower Davies–Bouldin index than k = 4 or k = 5 but produced a singleton group and highly unequal group sizes (1, 6, 9, 13, 16, and 21). After the compprehensively considering various results and setting k = 6 as the sampling solution, one-to-one candidate-to-centroid distances ranged from 0.001 to 3.269, with St. John’s Cathedral showing the largest distance.

### 4.3 Subjective restoration, affect, and cognitive performance

Perceived restorativeness differed significantly across the six scenes (χ^2^(5) = 103.67, *p* < 0.001, q < 0.001). Oil Street Art Space received the highest score (5.69/7; 95% CI 5.55–5.84), followed by Old Wan Chai Post Office (5.11; 4.95–5.26), whereas Western Market received the lowest score (4.17; 4.00–4.33). The 1.52-point difference between the highest and lowest scenes represents approximately 22% of the seven-point scale, indicating a clear descriptive magnitude in addition to statistical significance. Among the secondary affective outcomes, stress, calmness, and positive affect also showed scene effects after correction, whereas tension was not significant after within-family FDR correction (Figure 13A).

**Figure 13:**
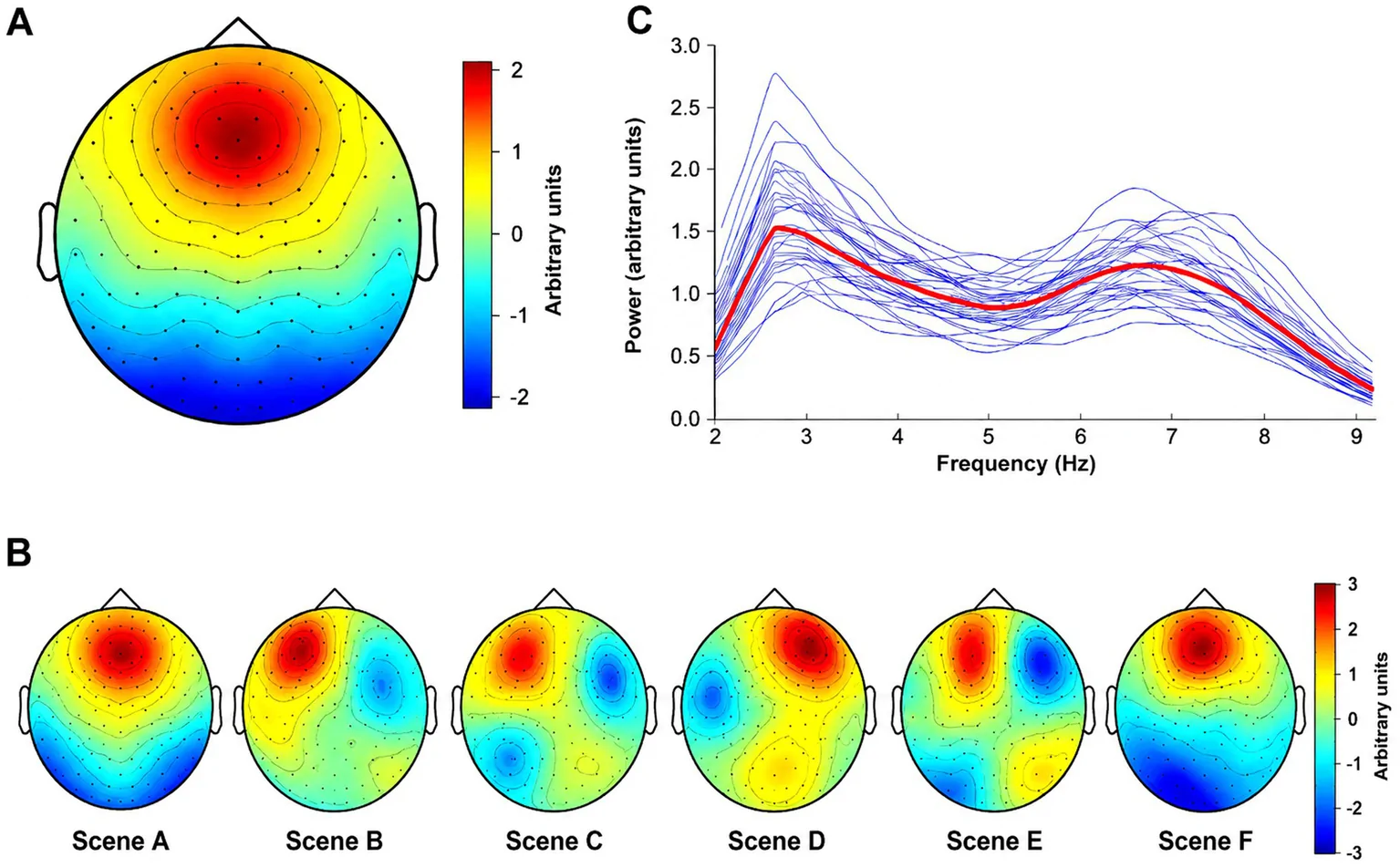
EEG Scalp Topography for 6 Scenes.

Sustained-attention accuracy did not differ significantly among scenes (χ^2^(5) = 5.64, *p* = 0.342, q = 0.342; Figure 13B), with descriptive values ranging from 78.9% at Hong Kong Science Museum to 87.5% at Oil Street Art Space. Thus, although the scenes produced clearly different ratings of restorativeness, participants maintained broadly similar accuracy in the short sustained-attention task. Correct-response reaction time, treated as a secondary outcome, did differ significantly (χ^2^(5) = 30.33, q < 0.001), with Oil Street Art Space producing the fastest responses (528.8 ms) and Hong Kong Museum of Art the slowest (579.3 ms). The resulting cognitive pattern was therefore one of variable response speed but relatively stable accuracy. This finding provides an important interpretive boundary: 60-s visual exposure was sufficient to alter subjective and physiological states but not necessarily the accuracy of short-term attentional performance.

### 4.4 Visual-attention responses

The scene effect was particularly pronounced for the proportion of fixations on natural AOIs (χ^2^(5) = 214.58, *p* < 0.001, q < 0.001). At Oil Street Art Space, 51.4% of AOI fixations fell on vegetation, water, or sky, compared with 42.9% at Old Wan Chai Post Office and only 31.2% at Western Market (Figure 13). The 20.2-percentage-point difference between Oil Street Art Space and Western Market coincided with the scenes showing the highest and lowest perceived restorativeness. This parallel differentiation provides descriptive cross-modal evidence that scenes rated as more restorative also attracted more visual attention toward nature-related AOIs.

Gaze entropy also differed significantly among scenes (χ^2^(5) = 25.01, *p* < 0.001, q < 0.001), but its ranking did not mirror perceived restorativeness. Hong Kong Museum of Art had the highest gaze entropy (3.39), whereas Hong Kong Science Museum had the lowest (2.95), a difference of 0.44. Mean fixation duration showed no significant scene effect after correction (χ^2^(5) = 2.64, q = 0.755) (Figure 10). Higher gaze entropy indicates that gaze was distributed more broadly across spatial regions; it does not imply longer viewing time or greater liking. In other words, Hong Kong Museum of Art elicited more extensive visual search without producing the highest restorativeness rating. This result indicates a complex relationship between visual complexity or exploratory viewing and restorative experience.

### 4.5 EEG and autonomic changes related to restoration

EEG alpha-minus-theta increased from the stress phase to the scene phase in all six conditions and differed significantly among scenes (χ^2^(5) = 69.58, *p* < 0.001, q < 0.001). Oil Street Art Space showed the largest mean increase (+2.68), followed by Old Wan Chai Post Office (+2.36), whereas Western Market showed the smallest (+1.93). The 0.75 log-power-unit difference between the highest and lowest scenes again aligned with the two extremes of perceived restorativeness. HRV RMSSD also exhibited a significant scene effect (χ^2^(5) = 20.78, q = 0.001), with Oil Street Art Space showing the largest increase (+11.37 ms). Mean EDA SCL change likewise differed among scenes (χ^2^(5) = 15.93, q = 0.008) (Figures 10, 14). These findings indicate that different tourist streetscapes elicited different neural and autonomic changes following stress.

**Figure 14:**
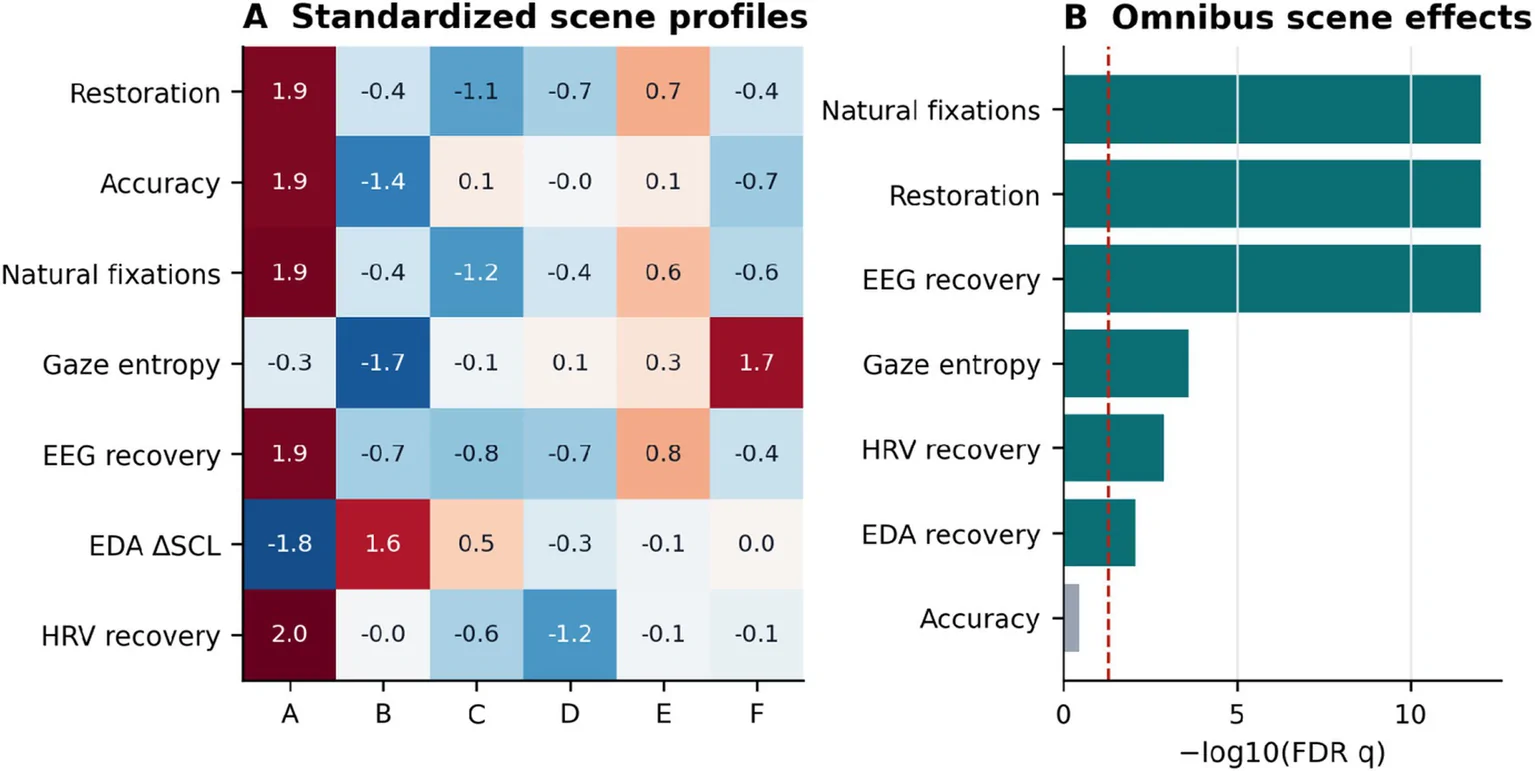
Standardised multimodal scene profiles and FDR-adjusted omnibus effects.

Quality control was implemented independently for each modality. Across the 108 focal trials, there was no evidence of deterioration with later presentation order in eye-tracking ratio (slope +0.00022 per order, *p* = 0.793), EEG artifact percentage (−0.132 percentage points per order, *p* = 0.164), or EDA valid-signal percentage (+0.026 percentage points per order, *p* = 0.796). Correct-response SART reaction time became modestly faster rather than slower across order (−2.49 ms per order, *p* = 0.021), which is compatible with a small practice/speeding effect and should not be interpreted as evidence against fatigue in other response channels. Scene × order interactions were not detected for perceived restorativeness (*p* = 0.583), natural-AOI fixation share (*p* = 0.543), gaze entropy (*p* = 0.162), or SART accuracy (*p* = 0.350), but were detected for EEG (*p* < 0.001), EDA (*p* = 0.030), and HRV (*p* = 0.013). The physiological scene contrasts should therefore be interpreted with greater caution as order-sensitive, rather than as order-invariant scene effects (Supplementary Table A5).

### 4.6 Comparative restorative outcomes across representative environments

Across the seven primary outcomes, the clearest descriptive pattern appeared at the high–low endpoints: Oil Street Art Space showed the highest perceived restorativeness and natural-AOI fixation share and the largest EEG and HRV increases, whereas Western Market showed the lowest perceived restorativeness, natural-AOI fixation share, and EEG increase. Sensitivity analyses nevertheless qualify this pattern. When only each participant’s earliest focal-scene exposure was retained (*n* = 46 independent observations; 45 for eye/EEG and 42 for HRV), scene differences remained for perceived restorativeness (*p* < 0.001), natural-AOI fixation share (*p* < 0.001), gaze entropy (*p* = 0.034), EEG (*p* < 0.001), and HRV (*p* = 0.040), but not for EDA (*p* = 0.527) or cognitive accuracy (*p* = 0.265). In addition, available-case versus 99-trial complete-case analyses yielded the same qualitative scene conclusions for EEG (both *p* < 0.001), EDA (*p* = 0.004 vs. 0.001), and HRV (both *p* < 0.001) (Supplementary Table A6).

Overall, the six fixed scenes displayed differentiated short-term response profiles rather than a single common restoration ranking across all modalities. Perceived restorativeness, natural-AOI fixation, and EEG/HRV changes showed partially concordant high–low patterns, whereas gaze entropy, EDA under the first-focal sensitivity restriction, and cognitive accuracy retained distinct information. Because physiological endpoints also showed scene-by-order interactions, the evidence supports robust subjective and attentional scene contrasts and more conditional physiological contrasts. These findings concern complete static visual scene configurations and do not identify a causal dose–response relationship for greenery, openness, visual order, or any other isolated feature.

## 5 Discussion

### 5.1 Main findings: restoration in high-density tourist space as a scene-configuration effect

The principal finding is that different high-density tourist scenes produced distinguishable short-term response profiles, but the strength of evidence differs by modality. Oil Street Art Space and Western Market formed clear descriptive endpoints for perceived restorativeness, natural-AOI fixation, and EEG change, and these contrasts persisted when analysis was restricted to each participant’s earliest focal-scene exposure. HRV also retained a scene effect in that sensitivity set, whereas EDA did not. Moreover, EEG, EDA, and HRV showed significant scene-by-order interactions in the full focal dataset. The physiological findings should therefore be read as order-sensitive scene contrasts.

This pattern argues against reducing restoration to a single environmental attribute. Oil Street Art Space and Old Wan Chai Post Office showed comparatively favorable response profiles despite occupying different positions in the objective PCA space. Their photographs jointly vary in color composition, visible natural elements, openness, architecture, heritage cues, signage, people, and visual organization.

A second finding is that restoration-related responses are stratified across time scales. A 60-s static visual exposure was sufficient to differentiate subjective restorativeness, EEG, EDA, and HRV, yet did not alter sustained-attention accuracy; only the secondary reaction-time outcome differed. ART and SRT should therefore not be interpreted as requiring simultaneous improvement across all indicators. A more plausible interpretation is that short visual exposure first alters scene appraisal, attentional allocation, and stress-related neural and autonomic states, whereas changes in cognitive performance may depend on longer exposure, task sensitivity, practice effects, and related conditions (7–14, 17).

Third, visual attention itself comprises distinct dimensions. The proportion of fixations on natural AOIs differentiated the high- and low-restorativeness endpoints in the same direction, whereas the highest gaze entropy occurred at Hong Kong Museum of Art rather than at the most restorative scene, Oil Street Art Space. This indicates that the proportion of gaze directed toward nature-related regions and the degree to which gaze is dispersed across the scene have different psychological meanings: the former characterizes attentional targets, whereas the latter characterizes the organization of visual search, and neither can be equated directly with preference. This distinction is particularly important for tourist streetscapes because landmarks, heritage details, commercial information, and wayfinding cues may all increase visual exploration without necessarily facilitating stress recovery. The value of multimodal measurement lies precisely in revealing this dissociation rather than collapsing all signals into a single score (30–32).

### 5.2 Urban-scale environmental heterogeneity and methodological contributions

Results from the 66 sites demonstrate pronounced multidimensional environmental variation within the high-density tourist core of Victoria Harbour. Built intensity, road structure, harbour proximity, and pedestrian-level green-, blue-, sky-color and image-structure proxies do not vary along a single high-to-low quality axis. PCA summarizes these correlated indicators into a smaller set of environmental gradients, providing a common coordinate system for transparent scene sampling. The clustering stage should be interpreted more cautiously: the alternative k solutions and the singleton at k = 6 show that the data do not support a uniquely defined six-type urban taxonomy.

The primary role of K-means in this study is therefore procedural. After comprehensively considering the results of several superior solutions, the study determined that k = 6 is the optimal numerical value. Alternative solutions were competitive on internal metrics, and the k = 6 solution contained one singleton group and one relatively distant candidate-to-centroid match.

Correlations among the deterministic color proxies further illustrate why measurement terminology matters. The blue and sky proxies were strongly correlated (ρ = 0.762), consistent with overlap in the blue-color information captured by the rules; Oil Street Art Space also had a high blue-color proxy that cannot be assumed to represent water. Natural-color saliency correlated with the green-color proxy, but neither relationship demonstrates semantic vegetation exposure or a causal restorative mechanism.

More importantly, this sampling framework provides a directly extensible pathway for future research: first define the environmental space at the urban scale; then prospectively photograph a medoid or rule-defined near-centroid scene from each stable archetype; and only thereafter proceed to the human-subject experiment. More credible feature-level effects can be estimated in multilevel models with both participant and scene random effects only when the number of scenes is substantially increased and collinearity among greenery, water/sky, heritage characteristics, enclosure, and visual complexity is deliberately reduced. The contribution of the present study is to demonstrate that high-density urban tourist attractions with different environmental characteristics can generate distinct, modality-specific profiles of restorative response.

Methodologically, this study connects two evidence streams that have often been examined separately within a single traceable chain: official sites and streetscape data first describe environmental heterogeneity at the urban scale; PCA/K-means then translate that heterogeneity into a principled basis for scene sampling; and field photographs are subsequently used in a participant experiment. The study also emphasizes the inherently multidimensional nature of multimodal environmental response. Perceived restorativeness captures subjective evaluation, eye tracking reflects attentional allocation, and EEG, EDA, and HRV represent different neural and autonomic processes with distinct temporal characteristics and data-quality requirements (53, 55, 56, 59). The modalities were therefore not standardized and summed into a putative composite streetscape quality index. Instead, the analysis tested whether different response channels partially converged at the scene level. The non-significant cognitive-accuracy result and the distinct gaze-entropy ranking provide essential boundaries for this multimodal interpretation.

### 5.3 Restorative microenvironments in tourist approach spaces and mapping restorative potential

For tourism-space research, the most direct implication is that approach streets, forecourts, and public spaces connecting transport nodes to attractions warrant attention as part of a sequential visitor experience, not merely as circulation corridors. This interpretation is consistent with visitor-generated studies showing that sensory experience along the Victoria Harbour waterfront differs by spatial context and user group (1), that destination image varies with length of stay and the content of posted visual/textual material (2), and that online reviews reveal both positive and negative dimensions of Hong Kong’s destination image (3). City-walk research further suggests that visual order participates in a dynamic cognitive-affective appraisal process during walking tourism (33). The present laboratory results add controlled short-term visual evidence to this broader field context but do not reproduce continuous walking, multisensory exposure, or real-time route choice.

The present data therefore support investigation of combinations of scene characteristics rather than optimization of isolated features. Hong Kong Museum of Art had the highest gaze entropy but was not the most restorative scene, showing that broader visual exploration alone is not equivalent to restoration. Old Wan Chai Post Office showed comparatively favorable restoration-related responses despite limited green-color proxy values, again indicating that multiple attributes co-occur within complete scenes. These contrasts motivate future factorial or larger-scene designs capable of separating greenery, openness, visual order, heritage cues, signage, and other correlated components.

On this basis, the study further proposes an environmental archetype–experimental response calibration methodology for assessing restorative potential in high-density tourist streetscapes. Specifically, the PCA/K-means archetypes derived from the 66 sites are used as an urban-scale spatial substrate; each archetype is linked to the multimodal response profile of its one-to-one matched field-photographed scene in perceived restorativeness, natural-AOI fixation, EEG, EDA, and HRV; and the experimentally calibrated archetype label is then projected back onto tourism sites belonging to the same class. Rather than mechanically combining multimodal outcomes into a single restoration index, the resulting map spatially stratifies key scene characteristics to identify relatively high-restorative-potential nodes, potentially low-restoration areas, and priority locations for intervention. Methodologically, this creates a spatial decision layer that links the objective environmental matrix, six environmental archetypes, and human experimental responses for use in tourism-space renewal and route organization.

### 5.4 Limitations and future research

This study has four principal limitations. First, k = 6 was a pragmatic six-scene sampling solution rather than a uniquely optimal clustering result. Second, each photograph contains multiple correlated visual attributes, and the deterministic color measures are proxies rather than validated semantic classes, preventing isolation of independent feature-level causal effects.

Third, 48 adults were registered but only 46 contributed at least one of the six focal-scene exposures analyzed here; the focal design is incomplete and unbalanced, and physiological endpoints showed significant scene-by-order interactions even though eye-tracking quality, EEG artifact percentage, and EDA valid-signal percentage did not deteriorate monotonically with order. Fourth, static two-dimensional visual exposure cannot reproduce walking, sound, heat, humidity, crowding, sequential route experience, or the full motivational context of tourism. These limitations require particular caution when translating laboratory physiological differences into mechanisms or planning recommendations.

Future research should return these scene-level findings to real approach routes by synchronously recording streetscape conditions, sound and thermal environments, crowding, walking behavior, and sequential exposure, while prospectively selecting a larger set of scenes that permits participant and scene random effects and feature-level contrasts. Urban-scale color proxies should be validated against manually annotated or independently validated semantic masks, and scene sampling should be repeated under alternative clustering or medoid-based strategies. Longitudinal or route-based studies could then test whether short-term visual differences predict dwell time, route choice, affective experience, and destination image under real tourist conditions.

## 6 Conclusion

This study established a multimodal experiment that links urban-scale tourist-streetscape sampling with controlled short-term visual measurement. Sixty-six official-source sites, 264 quality-checked streetscape images, and 11 objective environmental indicators first defined environmental heterogeneity in the high-density tourist core of Victoria Harbour. PCA reduced this information to principal environmental gradients. A six-group K-means solution was then used pragmatically to structure six-scene sampling, with alternative k diagnostics retained.

The strongest findings arise from the observed differences among the six complete scenes. Oil Street Art Space showed the highest perceived restorativeness (5.69/7), the highest natural-AOI fixation share (51.4%), and the largest EEG and HRV increases, whereas Western Market showed the lowest restorativeness (4.17/7), the lowest natural-AOI fixation share (31.2%), and the smallest EEG increase. These subjective and attentional contrasts were not explained by a detectable scene-by-order interaction and remained evident in the earliest-focal-exposure sensitivity analysis. Physiological contrasts were more conditional: EEG and HRV remained differentiated in the earliest-focal analysis, EDA did not, and all three physiological endpoints showed scene-by-order interaction in the full focal dataset.

Short-term restoration in high-density tourist space is therefore best interpreted here as a scene-configuration contrast rather than the linear effect of a single environmental indicator. Green-, blue-, and sky-color proxies, spatial openness, architectural and heritage characteristics, visual order, signage, and place meaning co-occur within each photograph. The present experiment shows that such complete configurations can produce differentiated subjective, attentional, neural, and autonomic response profiles, but it does not identify a causal dose–response mechanism for greenery, openness, legibility, or any other isolated feature (Figure 15).

**Figure 15:**
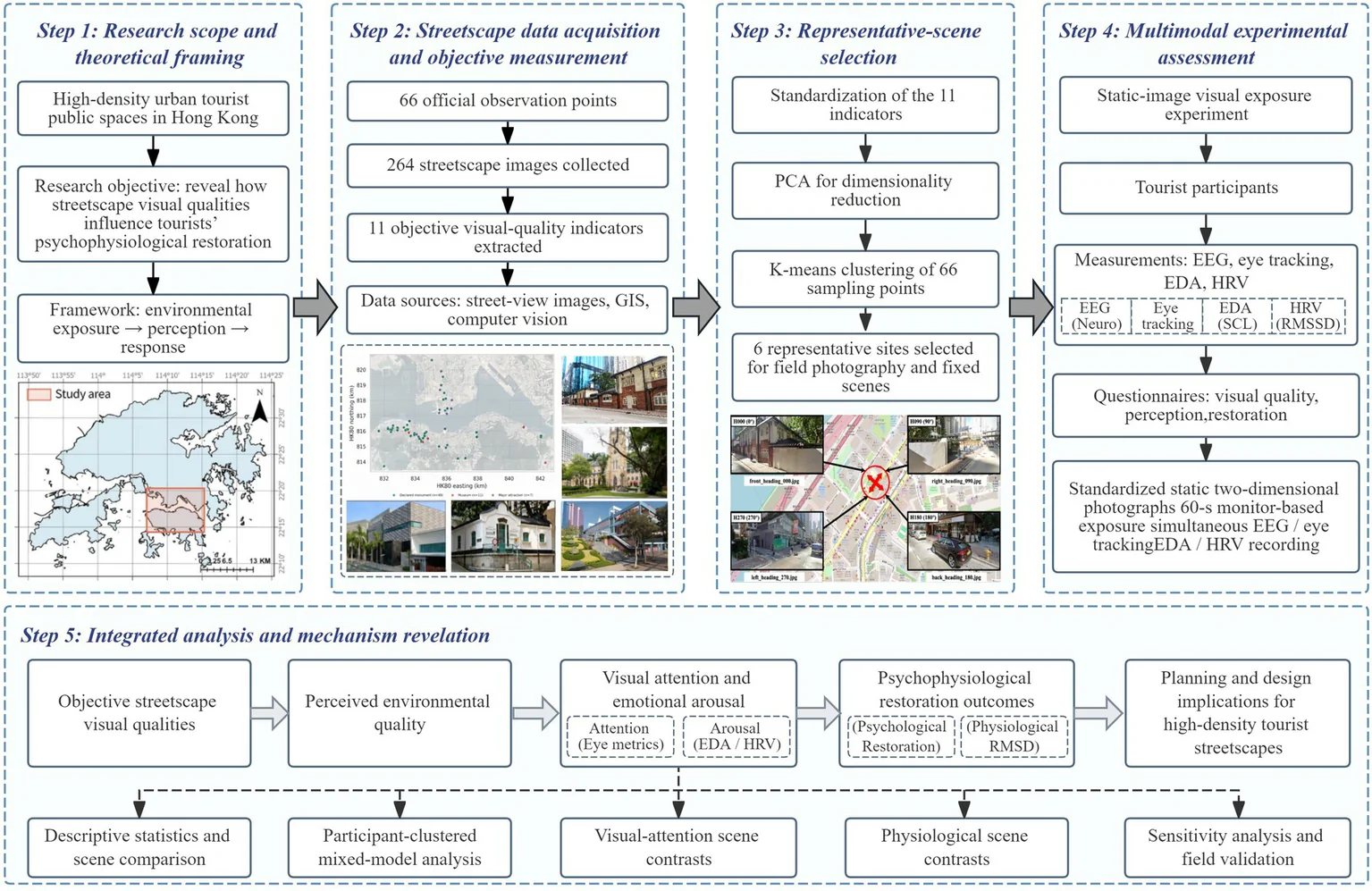
Research Workflow Design and cluster-guided scene selection.

For tourism and spatial design, these findings imply that approach streets, forecourts, and small public spaces around attractions can be reconceptualized as restorative infrastructure within the destination experience. High density does not inherently preclude restoration; within existing urban fabric, multiple spatial configurations with restorative potential may be created by retaining visible natural cues, providing moderately open and dwellable spaces, reducing disordered visual load, and strengthening heritage or landmark elements that support wayfinding and local meaning. Building on this principle, the study links the environmental archetypes of the 66 sites with the six-scene experimental responses and proposes an archetype-calibrated framework for mapping restorative potential in high-density tourist streetscapes. This extends restoration research from scene comparison to the identification of restorative opportunity nodes, diagnosis of potentially low-restoration areas, and prioritization of interventions. If these scene effects can be validated along real tourist routes, the approach could ultimately develop from point-based diagnosis toward a restorative route network for high-density destinations.

Future research should use a larger, prospectively selected set of scenes, complete standardized field photography based on stable environmental archetypes before participant recruitment, and include both participant and scene effects in the statistical models. Such an extension is necessary to determine which actionable combinations of spatial features genuinely drive tourist restoration and whether short-term psychophysiological effects translate into longer-term outcomes such as dwell time, route choice, and destination experience.

## Data Availability

The original contributions presented in the study are included in the article/Supplementary Material, further inquiries can be directed to the corresponding author.
