# Supplementary material for: Restorative streetscapes in high-density urban tourism: environmental archetypes, visual attention, and psychophysiological responses
Source: Front Public Health. 2026 Oct 22;14:1981997. doi: 10.3389/fpubh.2026.1981997 (PMC13736612; doi:10.3389/fpubh.2026.1981997)
Supplement: Supplementary file 1 [file Table_1.docx]

Supplementary Material

# Supplementary Tables

**Table A1.** Complete 66-site × 11-indicator matrix for PCA and clustering

| **ID** | **Official site** | **BCR** | **BVD (m)** | **Height (m)** | **RD (m ha⁻¹)** | **Harbour dist. (m)** | **Green** | **Blue** | **Sky** | **Nat. saliency** | **Entropy** | **Image quality** |
| --- | --- | --- | --- | --- | --- | --- | --- | --- | --- | --- | --- | --- |
| 0001 | Block 10 of the old Lei Yue Mun Barracks | 0.065 | 0.585 | 13.14 | 0.0 | 541.8 | 0.137 | 0.032 | 0.020 | 0.082 | 7.349 | 0.922 |
| 0002 | Block 7 of the old Lei Yue Mun Barracks | 0.065 | 0.585 | 16.09 | 0.0 | 475.4 | 0.070 | 0.048 | 0.053 | 0.060 | 7.461 | 0.910 |
| 0003 | Bonham Road Government Primary School | 0.462 | 13.652 | 31.40 | 221.1 | 3050.8 | 0.130 | 0.016 | 0.035 | 0.077 | 7.478 | 0.919 |
| 0004 | Chinese YMCA of Hong Kong | 0.363 | 10.899 | 29.91 | 236.0 | 3560.7 | 0.053 | 0.036 | 0.031 | 0.044 | 7.750 | 0.964 |
| 0005 | Duddell Street Steps and Gas Lamps | 0.335 | 16.172 | 37.43 | 262.5 | 4203.3 | 0.020 | 0.099 | 0.152 | 0.070 | 7.599 | 0.891 |
| 0006 | Eliot Hall, The University of Hong Kong (Exterior declared as a monument) | 0.365 | 7.298 | 29.59 | 64.6 | 2546.2 | 0.151 | 0.085 | 0.064 | 0.114 | 7.478 | 0.903 |
| 0007 | Facade of the Old Mental Hospital | 0.462 | 13.652 | 30.10 | 221.1 | 3033.4 | 0.025 | 0.016 | 0.044 | 0.026 | 7.496 | 0.908 |
| 0008 | Former Central Magistracy | 0.545 | 18.325 | 34.77 | 260.7 | 3984.9 | 0.055 | 0.022 | 0.038 | 0.042 | 7.834 | 0.956 |
| 0009 | Former Central Police Station Compound | 0.545 | 18.325 | 33.39 | 260.7 | 3966.9 | 0.068 | 0.036 | 0.043 | 0.053 | 7.825 | 0.967 |
| 0010 | Former French Mission Building | 0.421 | 25.915 | 44.72 | 180.5 | 4496.8 | 0.025 | 0.066 | 0.038 | 0.040 | 7.187 | 0.886 |
| 0011 | Former Kowloon British School | 0.506 | 13.802 | 25.25 | 92.1 | 6469.5 | 0.172 | 0.028 | 0.017 | 0.098 | 7.267 | 0.960 |
| 0012 | Former Marine Police Headquarters Compound | 0.595 | 16.025 | 27.44 | 316.4 | 5980.7 | 0.036 | 0.078 | 0.072 | 0.056 | 7.433 | 0.930 |
| 0013 | Former Victoria Prison Compound | 0.545 | 18.325 | 32.68 | 260.7 | 3944.2 | 0.004 | 0.017 | 0.017 | 0.010 | 7.404 | 0.933 |
| 0014 | Fung Ping Shan Building, The University of Hong Kong (Exterior declared as a monument) | 0.429 | 13.931 | 31.32 | 78.7 | 2538.5 | 0.030 | 0.070 | 0.077 | 0.051 | 7.317 | 0.878 |
| 0015 | Government House | 0.421 | 25.915 | 27.43 | 180.5 | 4253.2 | 0.063 | 0.130 | 0.114 | 0.093 | 7.644 | 0.932 |
| 0016 | Hong Kong City Hall | 0.293 | 11.989 | 32.59 | 358.3 | 4737.0 | 0.007 | 0.034 | 0.064 | 0.026 | 7.223 | 0.909 |
| 0017 | Hong Kong Observatory | 0.506 | 13.802 | 26.43 | 92.1 | 6488.0 | 0.020 | 0.030 | 0.027 | 0.024 | 7.705 | 0.913 |
| 0018 | Hung Hing Ying Building, The University of Hong Kong (Exterior declared as a monument) | 0.501 | 14.364 | 30.75 | 290.0 | 2466.4 | 0.022 | 0.127 | 0.131 | 0.075 | 7.575 | 0.935 |
| 0019 | Jamia Mosque | 0.669 | 24.753 | 34.03 | 173.6 | 3713.4 | 0.033 | 0.043 | 0.043 | 0.038 | 7.625 | 0.944 |
| 0020 | King Yin Lei | 0.181 | 3.806 | 19.04 | 149.3 | 5789.8 | 0.028 | 0.048 | 0.077 | 0.044 | 7.253 | 0.862 |
| 0021 | King's College | 0.719 | 22.267 | 30.81 | 182.4 | 2709.0 | 0.179 | 0.055 | 0.046 | 0.115 | 7.457 | 0.873 |
| 0022 | Kom Tong Hall | 0.622 | 22.897 | 32.18 | 189.7 | 3639.0 | 0.015 | 0.055 | 0.058 | 0.035 | 7.502 | 0.905 |
| 0023 | Kowloon Union Church | 0.467 | 10.944 | 25.70 | 333.9 | 6563.2 | 0.135 | 0.019 | 0.028 | 0.079 | 7.553 | 0.961 |
| 0024 | Kwong Fook Tsz | 0.363 | 10.899 | 31.03 | 236.0 | 3416.7 | 0.214 | 0.084 | 0.044 | 0.141 | 7.595 | 0.944 |
| 0025 | Lin Fa Temple | 0.295 | 7.463 | 26.16 | 258.5 | 4243.9 | 0.013 | 0.103 | 0.070 | 0.052 | 7.581 | 0.951 |
| 0026 | Lo Pan Temple | 0.476 | 17.925 | 34.57 | 118.3 | 1845.0 | 0.085 | 0.063 | 0.056 | 0.072 | 7.664 | 0.956 |
| 0027 | Lui Seng Chun | 0.442 | 11.400 | 24.67 | 376.2 | 5972.4 | 0.006 | 0.022 | 0.035 | 0.016 | 7.463 | 0.920 |
| 0028 | Main Building of St. Stephen's Girls' College | 0.462 | 13.652 | 32.63 | 221.1 | 2983.9 | 0.022 | 0.033 | 0.038 | 0.028 | 7.584 | 0.946 |
| 0029 | Main Building, The Helena May (Exterior declared as a monument) | 0.336 | 6.294 | 22.45 | 408.3 | 4352.0 | 0.038 | 0.054 | 0.025 | 0.040 | 7.168 | 0.881 |
| 0030 | Main Building, The University of Hong Kong (Exterior declared as a monument) | 0.501 | 14.364 | 30.02 | 290.0 | 2441.9 | 0.028 | 0.087 | 0.071 | 0.055 | 7.620 | 0.951 |
| 0031 | Man Mo Temple Compound | 0.605 | 20.734 | 29.95 | 204.0 | 3630.7 | 0.034 | 0.031 | 0.033 | 0.033 | 7.781 | 0.955 |
| 0033 | North and West Blocks of St. Joseph's College | 0.336 | 6.294 | 24.45 | 408.3 | 4358.6 | 0.015 | 0.042 | 0.059 | 0.032 | 7.419 | 0.949 |
| 0034 | Old Pathological Institute | 0.363 | 10.899 | 30.55 | 236.0 | 3460.3 | 0.254 | 0.048 | 0.052 | 0.152 | 7.512 | 0.932 |
| 0035 | Old Supreme Court (Exterior declared as a monument) | 0.293 | 11.989 | 53.80 | 358.3 | 4589.1 | 0.016 | 0.052 | 0.051 | 0.034 | 7.637 | 0.949 |
| 0036 | Old Wan Chai Post Office | 0.665 | 28.522 | 31.75 | 322.8 | 5864.8 | 0.059 | 0.037 | 0.030 | 0.047 | 7.091 | 0.868 |
| 0037 | Signal Tower at Blackhead Point | 0.401 | 21.047 | 31.28 | 337.6 | 6232.1 | 0.017 | 0.096 | 0.181 | 0.074 | 7.608 | 0.969 |
| 0038 | St. John's Cathedral | 0.421 | 25.915 | 34.65 | 180.5 | 4473.6 | 0.082 | 0.070 | 0.064 | 0.075 | 7.765 | 0.964 |
| 0039 | Tang Chi Ngong Building, The University of Hong Kong (Exterior declared as a monument) | 0.429 | 13.931 | 31.55 | 78.7 | 2620.7 | 0.098 | 0.162 | 0.086 | 0.115 | 7.580 | 0.929 |
| 0040 | The 21-Arch Section of the Bowen Aqueduct | 0.082 | 2.202 | 25.92 | 64.4 | 5479.9 | 0.036 | 0.026 | 0.051 | 0.036 | 7.226 | 0.880 |
| 0041 | The Bethanie | 0.281 | 7.678 | 13.39 | 83.1 | 2409.5 | 0.073 | 0.025 | 0.015 | 0.047 | 7.249 | 0.853 |
| 0042 | The Cenotaph | 0.293 | 11.989 | 47.66 | 358.3 | 4620.3 | 0.026 | 0.012 | 0.017 | 0.020 | 7.225 | 0.964 |
| 0043 | The Main Building of Old Tsan Yuk Maternity Hospital | 0.589 | 17.280 | 29.93 | 292.5 | 2810.0 | 0.033 | 0.034 | 0.042 | 0.035 | 7.330 | 0.865 |
| 0044 | Tin Hau Temple and the adjoining buildings | 0.360 | 8.670 | 24.99 | 496.9 | 6404.5 | 0.044 | 0.113 | 0.076 | 0.071 | 7.515 | 0.907 |
| 0045 | Tin Hau Temple, Causeway Bay | 0.589 | 20.875 | 32.47 | 243.6 | 4241.0 | 0.048 | 0.115 | 0.115 | 0.081 | 7.518 | 0.953 |
| 0046 | Tung Lin Kok Yuen | 0.259 | 8.202 | 26.73 | 180.9 | 5325.8 | 0.080 | 0.053 | 0.016 | 0.059 | 7.388 | 0.807 |
| 0047 | Tung Wah Museum | 0.359 | 12.946 | 30.60 | 199.2 | 6573.5 | 0.018 | 0.053 | 0.082 | 0.041 | 7.775 | 0.979 |
| 0048 | Western Market | 0.702 | 30.132 | 37.66 | 387.8 | 3769.2 | 0.023 | 0.045 | 0.057 | 0.036 | 7.258 | 0.868 |
| 0049 | Yuk Hui Temple (Wan Chai) | 0.427 | 15.331 | 30.78 | 161.6 | 5922.0 | 0.092 | 0.012 | 0.016 | 0.053 | 7.500 | 0.932 |
| 0050 | Avenue of Stars | 0.149 | 6.083 | 22.81 | 0.0 | 6201.8 | 0.071 | 0.074 | 0.085 | 0.075 | 7.339 | 0.876 |
| 0051 | Clock Tower | 0.292 | 9.488 | 16.58 | 0.0 | 5863.0 | 0.086 | 0.049 | 0.018 | 0.061 | 7.184 | 0.754 |
| 0052 | Golden Bauhinia Square (and Hong Kong Convention and Exhibition Centre) | 0.420 | 27.698 | 9.78 | 217.8 | 5963.0 | 0.015 | 0.089 | 0.063 | 0.047 | 7.583 | 0.880 |
| 0053 | Ladies' Market | 0.593 | 19.014 | 30.84 | 288.7 | 6497.4 | 0.046 | 0.034 | 0.036 | 0.040 | 7.481 | 0.939 |
| 0054 | Lan Kwai Fong | 0.666 | 36.774 | 38.00 | 284.5 | 4107.8 | 0.044 | 0.028 | 0.045 | 0.039 | 7.643 | 0.929 |
| 0055 | Temple Street Night Market | 0.538 | 15.446 | 24.56 | 335.8 | 6321.7 | 0.025 | 0.060 | 0.040 | 0.039 | 7.321 | 0.927 |
| 0056 | Tsim Sha Tsui Promenade | 0.000 | 0.000 | 28.80 | 21.0 | 5999.8 | 0.028 | 0.152 | 0.136 | 0.087 | 7.705 | 0.943 |
| 0057 | Conservation Resource Centre | 0.548 | 12.717 | 24.58 | 315.5 | 6122.9 | 0.019 | 0.019 | 0.033 | 0.022 | 7.421 | 0.841 |
| 0058 | Dr Sun Yat-sen Museum | 0.622 | 22.897 | 31.79 | 189.7 | 3636.2 | 0.031 | 0.070 | 0.044 | 0.045 | 7.537 | 0.889 |
| 0059 | Flagstaff House Museum of Tea Ware | 0.354 | 19.502 | 42.01 | 602.6 | 4772.0 | 0.021 | 0.077 | 0.111 | 0.056 | 7.678 | 0.943 |
| 0060 | Hong Kong Film Archive | 0.364 | 11.868 | 33.93 | 404.8 | 1213.9 | 0.387 | 0.079 | 0.071 | 0.232 | 7.764 | 0.967 |
| 0061 | Hong Kong Museum of Art | 0.278 | 7.472 | 22.68 | 1.1 | 6093.4 | 0.025 | 0.042 | 0.047 | 0.034 | 7.418 | 0.857 |
| 0062 | Hong Kong Museum of History | 0.548 | 12.717 | 24.58 | 315.5 | 6122.9 | 0.074 | 0.140 | 0.039 | 0.087 | 7.673 | 0.971 |
| 0063 | Hong Kong Science Museum | 0.548 | 12.717 | 24.77 | 315.5 | 6075.5 | 0.055 | 0.113 | 0.153 | 0.092 | 7.816 | 0.919 |
| 0064 | Hong Kong Space Museum | 0.598 | 21.945 | 25.48 | 305.0 | 6112.8 | 0.019 | 0.055 | 0.042 | 0.035 | 7.478 | 0.874 |
| 0065 | Hong Kong Visual Arts Centre | 0.336 | 6.294 | 25.02 | 408.3 | 4442.2 | 0.023 | 0.068 | 0.081 | 0.048 | 7.561 | 0.934 |
| 0066 | Law Uk Folk Museum | 0.509 | 18.094 | 32.66 | 220.3 | 2237.7 | 0.065 | 0.022 | 0.029 | 0.045 | 7.128 | 0.884 |
| 0067 | Oil Street Art Space | 0.500 | 27.306 | 48.61 | 225.8 | 4249.2 | 0.019 | 0.465 | 0.105 | 0.170 | 7.580 | 0.927 |

**Table A2.** PCA/K-means settings and internal validation for cluster-guided sampling

| **k** | **Silhouette** | **Calinski–Harabasz** | **Davies–Bouldin** | **Inertia** | **Minimum cluster size** |
| --- | --- | --- | --- | --- | --- |
| 2 | 0.268 | 16.91 | 1.389 | 460.22 | 13 |
| 3 | 0.241 | 18.46 | 1.451 | 366.82 | 11 |
| 4 | 0.254 | 19.11 | 1.246 | 302.33 | 6 |
| 5 | 0.220 | 18.82 | 1.303 | 260.45 | 6 |
| 6 | 0.226 | 18.51 | 1.092 | 228.84 | 1 |
| 7 | 0.233 | 18.80 | 1.016 | 199.83 | 1 |
| 8 | 0.230 | 18.76 | 0.924 | 178.24 | 1 |

**Table A3.** Candidate-site matching to six centroids in five-PC space

| **Scene** | **Site** | **Matched archetype** | **Hard nearest cluster** | **Matched centroid distance** | **PC1** | **PC2** | **Archetype size** |
| --- | --- | --- | --- | --- | --- | --- | --- |
| A | Oil Street Art Space | A | A | 0.000 | 5.198 | 1.529 | 1 |
| B | Hong Kong Science Museum | B | B | 1.592 | 2.019 | 0.560 | 13 |
| C | Western Market | C | C | 2.732 | 0.542 | -2.732 | 21 |
| D | St. John's Cathedral | D | B | 3.269 | 1.800 | 0.041 | 6 |
| E | Old Wan Chai Post Office | E | E | 2.899 | -0.603 | -2.453 | 16 |
| F | Hong Kong Museum of Art | F | F | 1.318 | -2.637 | 0.288 | 9 |

**Table A4**. Full focal-scene presentation-order distribution and modality-specific missingness.

| **Scene** | **n** | **Counts at presentation orders** | **Eye missing** | **EEG missing** | **EDA missing** | **HRV missing** |
| --- | --- | --- | --- | --- | --- | --- |
| A | 17 | 1/1/1/2/0/2/4/3/3/0 | 0 | 0 | 0 | 0 |
| B | 16 | 1/2/3/1/1/2/1/2/3/0 | 0 | 0 | 0 | 0 |
| C | 22 | 1/5/2/1/4/0/2/3/2/2 | 0 | 0 | 0 | 2 |
| D | 20 | 3/2/0/0/3/1/5/2/2/2 | 0 | 1 | 0 | 2 |
| E | 22 | 4/2/2/4/2/0/1/1/2/4 | 0 | 0 | 1 | 2 |
| F | 11 | 0/1/2/0/3/3/0/0/0/2 | 1 | 0 | 0 | 1 |

**Table A5.** Order-related sensitivity and signal-quality trend checks for the six focal scenes.

| **Check** | **Estimate / statistic** | **p value** | **Interpretation** |
| --- | --- | --- | --- |
| Scene × order: PRS | cluster-robust joint interaction test | 0.583 | No detected interaction |
| Scene × order: natural-AOI fixation share | cluster-robust joint interaction test | 0.543 | No detected interaction |
| Scene × order: gaze entropy | cluster-robust joint interaction test | 0.162 | No detected interaction |
| Scene × order: SART accuracy | cluster-robust joint interaction test | 0.350 | No detected interaction |
| Scene × order: EEG alpha-minus-theta change | cluster-robust joint interaction test | <0.001 | Order-sensitive scene contrast |
| Scene × order: EDA mean-SCL change | cluster-robust joint interaction test | 0.030 | Order-sensitive scene contrast |
| Scene × order: HRV RMSSD change | cluster-robust joint interaction test | 0.013 | Order-sensitive scene contrast |
| Eye-tracking ratio vs order | +0.00022 per order | 0.793 | No deterioration |
| EEG artifact percentage vs order | −0.132 percentage points per order | 0.164 | No deterioration |
| EDA valid-signal percentage vs order | +0.026 percentage points per order | 0.796 | No deterioration |
| Correct-response SART RT vs order | −2.49 ms per order | 0.021 | Modest speeding/practice pattern |

**Table A6**. Earliest-focal-exposure and complete-case sensitivity analyses.

| **Endpoint** | **Earliest focal N** | **Earliest focal scene p** | **Available-case N / p** | **Complete-case N / p** | **Sensitivity interpretation** |
| --- | --- | --- | --- | --- | --- |
| Perceived restoration | 46 | <0.001 | 108 / primary model significant | — | Scene contrast retained |
| Natural-AOI fixation share | 45 | <0.001 | 107 / primary model significant | — | Scene contrast retained |
| Gaze entropy | 45 | 0.034 | 107 / primary model significant | — | Scene contrast retained |
| SART accuracy | 46 | 0.265 | 108 / primary model not significant | — | No robust scene effect |
| EEG alpha-minus-theta change | 45 | <0.001 | 107 / <0.001 | 99 / <0.001 | Scene contrast retained; order interaction present |
| EDA mean-SCL change | 46 | 0.527 | 107 / 0.004 | 99 / 0.001 | Complete-case robust, but earliest-focal contrast not retained |
| HRV RMSSD change | 42 | 0.040 | 101 / <0.001 | 99 / <0.001 | Scene contrast retained; order interaction present |
